# Transcriptional regulation of stilbene synthases in grapevine germplasm differentially susceptible to downy mildew

**DOI:** 10.1186/s12870-019-2014-5

**Published:** 2019-09-14

**Authors:** Mario Ciaffi, Anna Rita Paolacci, Marco Paolocci, Enrica Alicandri, Valentina Bigini, Maurizio Badiani, Massimo Muganu

**Affiliations:** 1 Dipartimento per la Innovazione nei Sistemi Biologici, Agroalimentari e Forestali, Università della Tuscia, Via S. Camillo De Lellis, s.n.c, I-01100 Viterbo, Italy; 2 Dipartimento di Scienze Agrarie e Forestali, Università della Tuscia, Via S. Camillo De Lellis, s.n.c, I-01100 Viterbo, Italy; 3 Dipartimento di Agraria, Università Mediterranea di Reggio Calabria, Loc. Feo di Vito, I-89129 Reggio Calabria, Italy

**Keywords:** Chalcone synthase, Downy mildew susceptibility, Grapevine germplasm, MYB14 and MYB15, Plant-pathogen interactions, *Plasmopara viticola*, Stilbene synthase, *Vitis vinifera* L

## Abstract

**Background:**

To limit the impact of the downy mildew disease of grapevine and reduce the need to recur to chemical treatments, an effective strategy might be recovering adaptive resistance traits in both cultivated and wild *V. vinifera* germplasm*.* Considering that stilbenes represent the most important class of phytoalexins in the *Vitaceae*, the constitutive expression and transcriptional activation of all the functional members of the stilbene synthase gene family were analysed in a group of nine grapevine genotypes following artificial infection with the oomycete *Plasmopara viticola*, the causal agent of the disease. In addition, in the same genotypes we analyzed the expression of genes encoding for two transcription factors involved in the transcriptional regulation of the stilbene synthase genes, namely *VvMYB14* and *VvMYB15*, and of genes encoding for chalcone synthases.

**Results:**

Downy mildew incidence and severity ranged from nihil to high in the grapevine genotypes considered, being low to moderate in a subgroup of *V. vinifera* genotypes. The constitutive expression of the stilbene synthase genes as well as the extent of their transcriptional activation following *P. viticola* inoculation appeared to be inversely related to the proneness to develop disease symptoms upon infection. In a specular manner, following *P. viticola* inoculation all the chalcone synthase genes were up-regulated in the susceptible grapevine genotypes and down-regulated in the resistant ones. The infection brought by *P. viticola* appeared to elicit a co-ordinated and sequential transcriptional activation of distinct stilbene synthase genes subsets, each of which may be regulated by a distinct and specific MYB transcription factor.

**Conclusions:**

The present results suggest that the induction of stilbene biosynthesis may contribute to the basal immunity against the downy mildew of grapevine, thus representing an adaptive resistance trait to recover, in both cultivated and wild *V. vinifera* germplasm. During the early stages of *P. viticola* infection, an antagonistic interaction between flavonol and stilbene biosynthesis might occur, whose outcome might determine the subsequent extent of disease symptoms. Further studies are needed to decipher the possible regulatory mechanisms involved in the antagonistic crosstalk between these two metabolic pathways in resistant and susceptible genotypes in response to *P. viticola*.

## Background

European grapevine (*Vitis vinifera L.*) is one of the most extensively cultivated plant, whose economic importance is witnessed by an almost seven and a half million Ha coverage worldwide [1]. Spain, China, France and Italy are the Countries with the largest vineyard areas [1]. Cultivated grape varieties are greatly affected by different destructive diseases, among which downy mildew, which can be considered the most severe one in the cultivation areas where mild temperatures, high humidity and abundant spring rainfalls are present [2].

The causal agent of the downy mildew of grapevine, i.e. *Plasmopara viticola* (Berk. et Curt.) Berl. et De Toni (PV), is a biotrophic obligatory oomycete which, in order to complete its life cycle, has to obtain nutrients from the living cells of its host. Such pathogen was fortuitously introduced in France from North America during the nineteenth century and rapidly spread across Europe [3]. It infects all green parts of the plant, causing, under favourable weather conditions, extensive losses in grape yield [4]. The damages caused by the pathogen can lead both to quantitative losses, by infecting inflorescences and bunches, and to qualitative decay, by causing an early defoliation of the plant [5, 6].

The potential harm by the pathogen, combined with a low efficacy of the agronomic practices to combat it, requires regular application of fungicides. However, the intensive use of chemicals is becoming more and more restricted, due to the risk for human health and the negative impact on the environment [7]. The European Directive 2009/128/EC establishes a framework for Community action to achieve the sustainable use of pesticides. One of the key features of such Directive is that each Member State should develop and adopt its National Action Plan and set up quantitative objectives, targets, measures and timetables to reduce risks and impacts of pesticides on human health and the environment. Moreover, the Directive encourages the development and introduction of integrated pest management and of alternative approaches or techniques, in order to reduce dependency on the use of pesticides.

To limit the impact of a plant pathogen, and reduce the need to recur to chemical treatments, the most effective strategy is the adoption of resistant plant material. In the case of the PV-*V. vinifera* pathosystem, this has been achieved by crossing *V. vinifera* with American wild species, such as *V. riparia, V. labrusca, V. aestivalis* and *V. berlandieri,* whose resistance to PV co-evolved with the pathogen in its place of origin [8]. The first generation hybrids, obtained more than one century ago, were unsuitable for the production of high quality wines, due to their unpleasant foxy aromas, coming from the American *Vitis* species, especially *V. labrusca* [8]. Nowadays, however, after many backcrossing cycles, the last generation breeding varieties possess both resistance to PV from the American species and desirable qualitative traits from *V. vinifera* [9].

An alternative strategy against PV, to which considerable less attention has been paid so far, could be recovering adaptive resistance traits from the *V. vinifera* germplasm, which includes cultivated (*V. vinifera* subsp. *vinifera)* and wild (*V. vinifera* subsp. s*ylvestris)* subspecies*,* originally dispersed from western Asia to Europe [10]. Indeed, several recent studies, run either in the field or in controlled environment, pointed out that cultivated *V. vinifera* varieties [11–17], including clones obtained from a single variety [18], as well as *V. vinifera* wild accessions [19, 20], exhibit a varying degree of susceptibility to PV.

The Italian *V. vinifera* germplasm includes several minor or local varieties whose level of resistance to biotic stresses, as well as viticultural and oenological characteristics, are still unknown. A detailed analysis of the variability in their susceptibility to PV, as well as of the underlying genetic and molecular defense mechanisms, could simplify the breeding programs and allow the selection of resistant grapevine varieties for the sustainable production of high quality wines.

In the *Vitaceae*, stilbenes represent the most important class of phytoalexins, which accumulate in response to a variety of environmental challenges, including pathogen attack [21–23]. The biosynthetic pathway leading to the production of stilbenes is a side branch of the general phenylpropanoid pathway and can be considered as an extension of the flavonoid pathway [24]. The key enzymes in the biosynthesis of stilbenic compounds are the stilbene synthases (STSs), which compete for the same substrates (*p*-coumaroyl-CoA and cinnamoyl-CoA) with chalcone synthases (CHSs), the key enzymes in the biosynthesis of flavonoids [25].

In agreement with the potential involvement of stilbenes in the plant response to pathogens, it has been shown that the expression of the genes encoding for STS in grapevine is strongly induced upon infection by PV [26–28]. Up-regulation of *STS* genes and other plant defense genes has been considered to contribute to the constitutive defense against pathogens during the development of grape berry [29].

The results demonstrating that *STS* genes are developmentally regulated and induced by infection of pathogens were often obtained by analyzing the *STS* genes as a group. Indeed, the annotation of the *V. vinifera* genome [30, 31] made it possible to identify up to 48 putative *VvSTS* gene sequences, with at least 32 of them encoding full-length proteins [26, 32]. The existence of multiple *STS* genes in the grapevine genome suggests, on one side, a prominent role of the STS-mediated pathways in the adaptation of this species to environmental challenges, and prompts, on the other side, to investigate the specific regulation of each individual *STS* gene in response to them.

During previous studies of ours on five *V. vinifera* varieties belonging to the oenological tradition of Central Italy, namely “Aleatico”, “Canaiolo nero”, “Romanesco”, “Rossetto”, and “Trebbiano toscano”, we found differences in the degree of resistance to PV, which could be putatively ascribed to a differential production of stilbenic compounds [5, 16]. To deepen our understanding of such differential responses in the aforementioned plant material, we analyzed here the temporal expression patterns of all the functional members of the *VvSTS* gene family during the first 72 h after artificial PV inoculation. In addition, genes encoding for two transcription factors involved in the transcriptional regulation of *STS* genes, namely *VvMYB14* and *VvMYB15* [33], as well as those encoding for chalcone synthases (*VvCHS*), were also considered here. To complete the array of differential responses to PV, and to evaluate the effectiveness of experimental inoculation, we included in the present comparison also “reference” grapevine varieties whose degree of susceptibility to downy mildew was a priori known: i.e., two hybrid cultivars, namely “Isabella” and “Solaris”, inherently resistant to PV (see above), and one PV-susceptible variety, namely “Chasselas”. Furthermore, looking for resistance traits to be found also outside of the cultivated grapevine germplasm, we evaluated here the responses to PV of a local wild accession of *V. vinifera* subsp. *sylvestris*.

## Results

### Downy mildew incidence and severity ranged from nihil to high in the selected group of grapevine genotypes

**Fig. 1:**
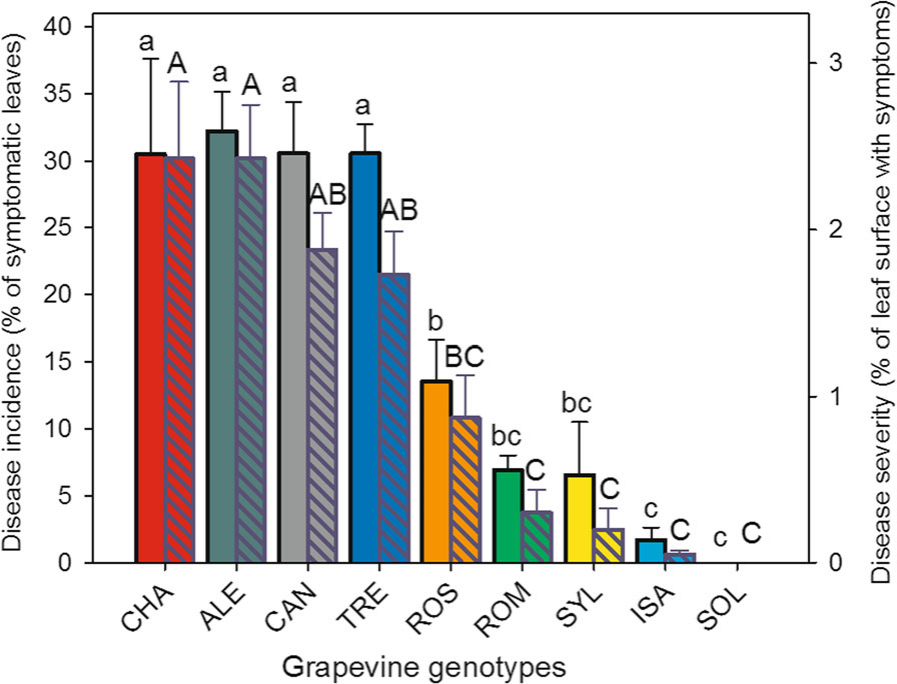
Mean values of disease incidence (plain bars, left axis, small letters) and severity (pattern bars, right axis, capital letters) evaluated in nine different *Vitis vinifera* genotypes 7 days after inoculation with *Plasmopara viticola*. Bars represent standard error of the mean. Different letters denote significant differences according to the Tukey’s test (*p* ≤ 0.05). Grapevine genotypes: CHA = Chasselas, ALE = Aleatico, CAN=Canaiolo nero, TRE = Trebbiano toscano, ROS = Rossetto, ROM = Romanesco, SYL = accession of *V. vinifera* subsp. *sylvestris,* ISA = Isabella, SOL = Solaris

In Fig. 1, the grapevine genotypes were ordered based on the incidence and severity of downy mildew visible symptoms 7 days after PV inoculation. The susceptible variety “Chasselas” (CHA) was one of the most infected genotypes, whereas, at the opposite end of the susceptibility ranking, the interspecific variety “Solaris” (SOL) did not show any areas of sporulation on its leaves and exhibited browning necrotic spots (Additional file 4: Figure S1), a hypersensitive reaction symptom already described in certain resistant grapevine genotypes infected by PV [12, 34, 35]. Differences were recorded in disease incidence among the five Italian *V. vinifera* varieties (Fig. 1, plain bars): “Aleatico” (ALE), “Trebbiano toscano” (TRE) and “Canaiolo nero” (CAN) showed significantly higher percentages of symptomatic leaves per plant, compared to “Rossetto” (ROS) and “Romanesco” (ROM). Downy mildew incidence in the wild grapevine accession (*V. vinifera* subsp. *sylvestris*, SYL) was among the lowest within the group of genotypes considered, and very similar, on one hand, to the least susceptible among the cultivated *V. vinifera* varieties, i.e. ROM and, on the other hand, to the first-generation hybrid “Isabella” (ISA) (Fig. 1, plain bars), which, even if not immune, was also confirmed to be highly resistant to PV.

Evaluating disease severity (Fig. 1, pattern bars) confirmed the presence of different degrees of susceptibility to downy mildew among the considered grapevine varieties, with ROS and ROM being the most resistant within the *V. vinifera* subgroup. In the latter, as well as in SYL, disease severity appeared to be as low as in the inherently resistant hybrids ISA and SOL (Fig. 1 pattern bars).

No downy mildew symptom was observed in control plants of each of the nine genotypes subjected to mock inoculation (data not shown).

On a symptomatic basis, in summary, the results in Fig. 1 allowed to divide the grapevine genotypes into three distinct groups: i) those highly susceptible to PV, represented by the *V. vinifera* varieties CHA, ALE, CAN and TRE, ii) the moderately susceptible ones, comprising the *V. vinifera* ROS and ROM, and the *V. sylvestris* accession (SYL), and iii) the resistant ISA and SOL hybrids; it has to be emphasized that Solaris was quite immune.

### Identification of gene sequences and synthesis of primers for expression analysis

#### Stilbene synthase (STS) genes

**Fig. 2:**
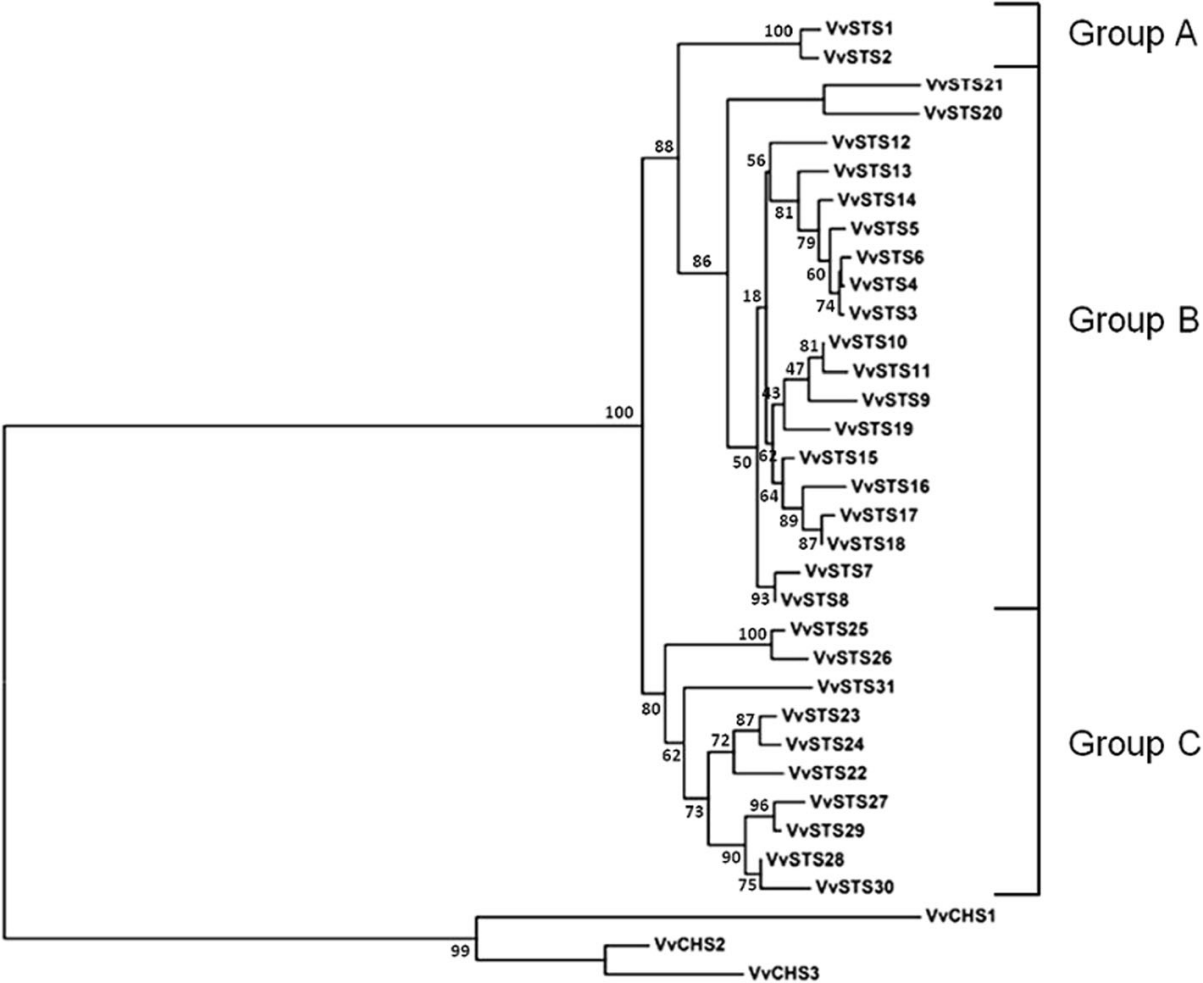
Phylogenetic tree of the deduced amino acid sequences of the *Vitis vinifera* stilbene synthase genes (*VvSTS*) identified in the NCBI database. The multiple alignment of 34 protein sequences (31 VvSTS and three *V. vinifera* chalcone synthases) was performed by ClustalX 1.83 software and the phylogenetic tree was constructed by the neighbor-joining (NJ) method and evaluated by bootstrap analysis (MEGA 7). The numbers on the main branches indicate bootstrap percentages for 1000 replicates. The three phylogenetic groups (A, B and C) identified in the grapevine *STS* family are highlighted with square brackets

The search in the NCBI database allowed us to identify 31 functional *STS* genes in the *V. vinifera* genome, all containing an ORF of 1179 bp coding for proteins of 392 amino acids in length (Additional file 1: Table S1). The 31 identified *STS* genes were named *VvSTS1–31* on the basis of their chromosomal location. *VvSTS1/2* are located on chromosome 10, whereas the remaining 29 (*VvSTS3–31*) form two distinct clusters in the central and distal regions of chromosome 16 (Additional file 1: Table S1). On the basis of phylogenetic analysis and comparisons between the nucleotide and amino acid sequences identified in the NCBI database and those previously characterized in the grapevine genome [27], it was possible to assign the 31 genes to the three phylogenetic groups into which the grapevine *VvSTS* family is divided (Fig. 2). Of the 31 functional *VvSTS* genes identified in the NCBI database, two (*VvSTS1* and *VvSTS2*), belong to the A group, 19 (*VvSTS3–21*) to the B group, and 10 (*VvSTS22–31*) to the C group (Fig. 2). The *VvSTS* genes located in the two distinct clusters of chromosome 16 showed a high level of similarity in their coding regions (nucleotide identity between 89 and 99%), indicating that they probably arose from segmental and tandem duplication events occurred in the *Vitaceae* lineage, after the separation from other dicots [27, 32]. Similarly, the alignment of the nucleotide sequences of the two *VvSTS* genes located on chromosome 10 showed a 99% identity among their coding regions.

Due to the complexity of the *VvSTS* gene family and the high level of conservation among most of their members (see above), only for eight out of the 31 genes identified, namely *VvSTS12, VvSTS13, VvSTS14, VvSTS15, VvSTS19, VvSTS20, VvSTS21* and *VvSTS31,* was it possible to design, from differences detected in the 3′ end region, specific primers for expression analysis (Additional file 1: Table S2). For the remaining 23 genes, nine pairs of conserved primers were designed (denoted with asterisks in Additional file 1: Table S2), each amplifying from two to four very similar sequences (referred to as *VvSTS1/2, VvSTS3/4, VvSTS5/6, VvSTS7/8, VvSTS9–11, VvSTS16–18, VvSTS22–24, VvSTS25/26, and VvSTS27–30* in the following).

#### Chalcone synthase (CHS) genes

The search in the NCBI database allowed us to identify five functional *CHS* genes in the *V. vinifera* genome, two of which, named *VvCHS2* and *VvCHS3*, located in chromosome 14, whereas each of the remaining three was located in a distinct chromosome: *VvCHS1* in chromosome 5, *VvCHS4* in chromosome 3 and *VvCHS5* in chromosome 15 (Additional file 1: Table S3). Specific primers were designed for analysing the expression of each *VvCHS* gene (Additional file 1: Table S3).

#### MYB transcription factors

The expression of genes encoding for two MYB transcription factors, namely VvMYB14 and VvMYB15, known to be involved in the transcriptional regulation of the *VvSTS* genes in response to biotic and abiotic stresses [33], were considered here. The known nucleotide sequences of *VvMYB14* (VIT_07s0005g03340) and *VvMYB15* (VIT_05s0049g01020) [33] were used to perform a search using the BLAST algorithm in the NCBI database. Then, the corresponding mRNA RefSeq sequences were used to design specific primers for each of the two genes (Additional file 1: Table S4).

### The expression level of most of the stilbene synthase genes before *P. viticola* inoculation was inversely related to the proneness to develop visible symptoms of downy mildew upon infection

**Fig. 3:**
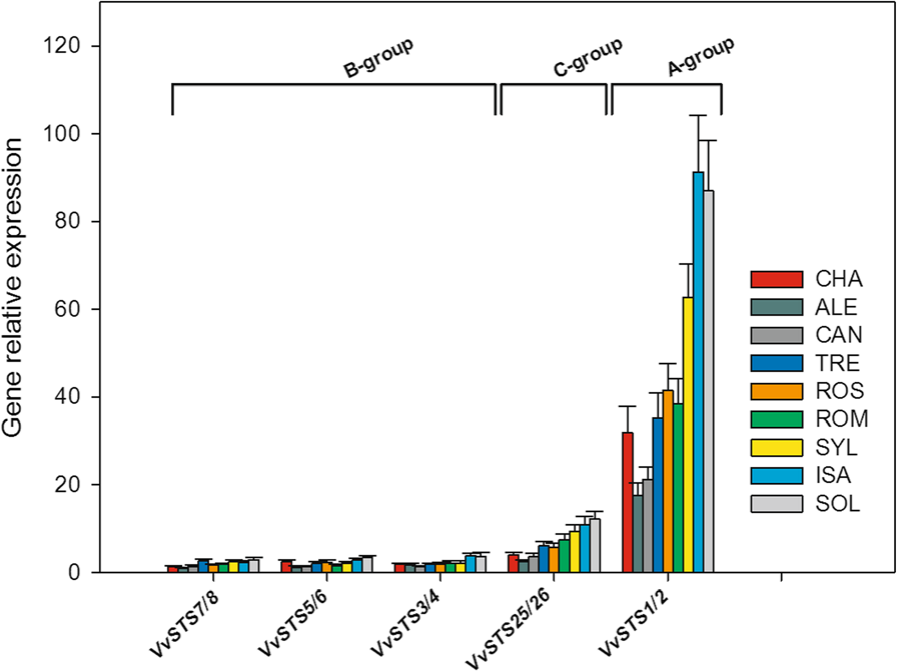
Relative expression levels of the group A *Vitis vinifera* stilbene synthase gene family members (*VvSTS1/2*) and of selected members of groups C and B in the leaves of the nine grapevine genotypes of Fig. 1 collected before inoculation with *Plasmopara viticola*. The expression data of each gene were normalized using the geometric average of the two reference genes *VvEF1α* and *VvSAND*. Relative expression levels of the different *VvSTS* genes were referred to a calibrator, set to the value 1, which was represented by the gene in the nine genotypes with the lowest expression (*VvSTS7/8* in the cv. Aleatico, ALE). Each reported value is the mean ± SD of three biological replicates, each of which was analyzed in triplicate. Grapevine genotypes: CHA = Chasselas, ALE = Aleatico, CAN=Canaiolo nero, TRE = Trebbiano toscano, ROS = Rossetto, ROM = Romanesco, SYL = accession of *V. vinifera* subsp. *sylvestris,* ISA = Isabella, SOL = Solaris

**Fig. 4:**
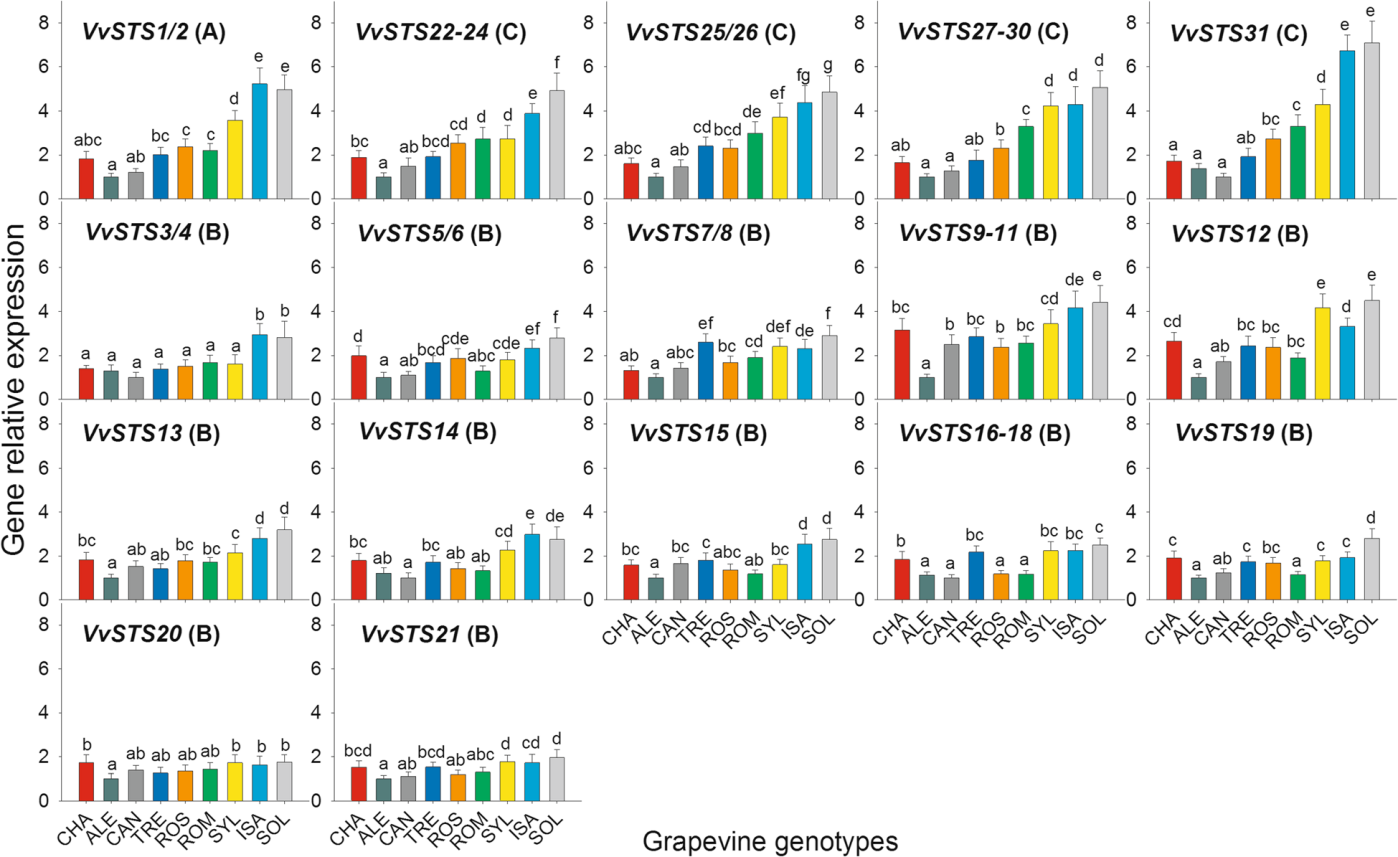
Relative expression levels of the whole set of the *Vitis vinifera* stilbene synthase genes (*VvSTS*) in the leaves of the nine grapevine genotypes of Fig. 1 collected before inoculation with *Plasmopara viticola*. The letter denoting the phylogenetic group to which each gene belongs is given in parentheses. For each gene, relative expression levels were calculated by setting a value of 1 for the lowest value among the nine genotypes. Normalization and sample replication as in Fig. 3. Different letters denote significant differences according to the Tukey’s test (*p* ≤ 0.01). Grapevine genotypes: CHA = Chasselas, ALE = Aleatico, CAN=Canaiolo nero, TRE = Trebbiano toscano, ROS = Rossetto, ROM = Romanesco, SYL = accession of *V. vinifera* subsp. *sylvestris,* ISA = Isabella, SOL = Solaris

Figure 3 shows the relative expression of selected members of the three phylogenetic groups of *VvSTS* gene family in the leaves of the grapevine genotypes collected before PV inoculation, whereas Fig. 4 presents the differences observed among the grapevine genotypes for the constitutive expression of each single or groups of *VvSTS* genes considered. Figure 3 puts into evidence that, regardless of the grapevine genotype considered, ample and significant differences were found among members of the three phylogenetic groups of *VvSTS* gene family in terms of constitutive expression. In particular, the A-group genes (*VvSTS1/2)* showed a transcription level that was 5–10 times and 10–20 times higher than that of members of the C and B groups, respectively (Fig. 3).

Figure 4 shows that ample and significant differences were present among the grapevine genotypes in terms of constitutive expression of *VvSTS* genes, mostly evident for the members of the A and C phylogenetic groups. It is also shown that, at least for the A and C groups, the levels of relative expression of the *VvSTS* genes before PV inoculation went in parallel with the degree of resistance/susceptibility to downy mildew observed thereafter (compare Fig. 4 and Fig. 1). In fact, and comparatively speaking, the amounts of transcripts of the A and C genes were higher in SOL, ISA, and in SYL, intermediate in ROM, ROS, TRE and CHA, and very low in CAN and ALE. However, it is worth noting that in the first group of genotypes the transcription levels of the A and C genes were almost always significantly higher in SOL and ISA than in SYL, and that, as regards the varieties with an intermediate level of expression, the *V. vinifera* cultivar ROM showed always significantly higher amounts of transcripts than the two PV-susceptible varieties CAN and ALE (Fig. 4).

As far as the B group of *VvSTS* genes is concerned, the differences in constitutive expression among the nine genotypes were less pronounced than for the A and C genes. Nevertheless, the constitutive expression of B genes in the SOL and ISA hybrids was most often significantly higher than in the remaining genotypes (Fig. 4). On the other hand, no significant differences in the level of constitutive expression of several B genes (such as *VvSTS3/4*, *VvSTS13*, *VvSTS14* and *VvSTS15*) were detected among the six *V. vinifera* varieties. Furthermore, no difference was apparent among the nine grapevine genotypes as far as the B genes *VvSTS20* and *VvSTS21* are considered (Fig. 4).

The constitutive expression of the *STS* genes in the mock-inoculated plants was found to be identical to that of the PV-inoculated ones (data not shown).

### Following *P. viticola* inoculation, the stilbene synthase genes showed distinct time profiles of transcripts accumulation, whose extent was proportional to the constitutive expression in the different grapevine genotypes

**Fig. 5:**
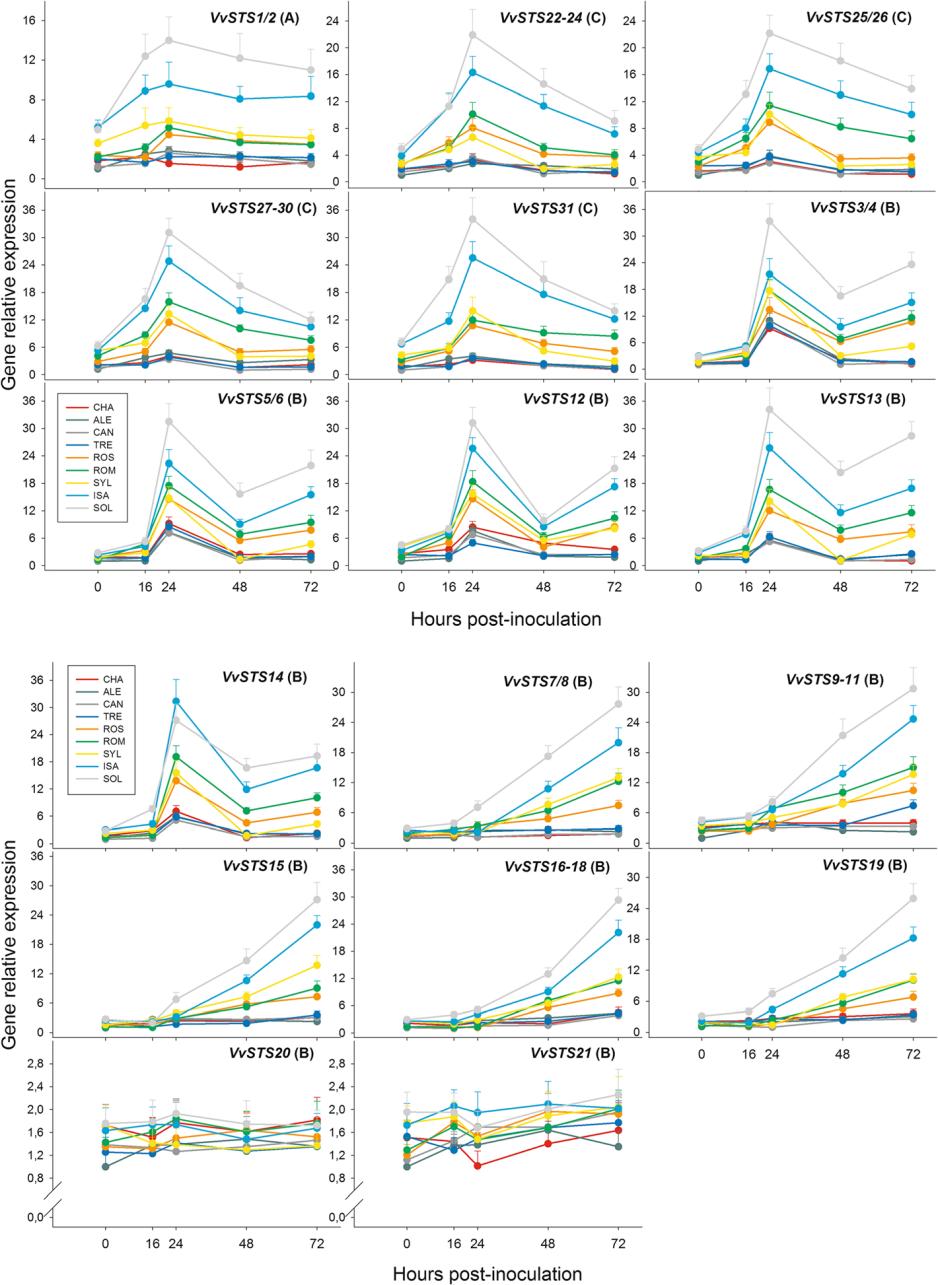
Relative expression levels of the whole set of the *Vitis vinifera* stilbene synthase genes (*VvSTS*) in the leaves of the nine grapevine genotypes of Fig. 1 collected up to 72 h after inoculation with *P. viticola*. The letter denoting the phylogenetic group to which each gene belongs is given in parentheses. For each gene, relative expression levels were calculated by setting a value of 1 for the lowest value among the nine genotypes in each of the five time points considered. Normalization and sample replication as in Fig. 3. For each gene, statistical evaluation of the differences among the nine grapevine genotypes and among the five sampling times is reported in Additional file 2: Table S6. Grapevine genotypes: CHA = Chasselas, ALE = Aleatico, CAN=Canaiolo nero, TRE = Trebbiano toscano, ROS = Rossetto, ROM = Romanesco, SYL = accession of *V. vinifera* subsp. *sylvestris,* ISA = Isabella, SOL = Solaris

Figure 5 and Additional file 2: Table S6 present the transcriptional time course of each component of the *VvSTS* gene family up to 72 h after inoculation (hpi) with *P. viticola*. Additional file 5: Figure S2 shows the time courses of gene expression in the form of heat maps. It is evident that the *VvSTS* members belonging to the three phylogenetic gene groups were differentially regulated upon inoculation with PV, and that, for each gene, ample and significant differences in the amounts of gene transcripts were present among the nine grapevine genotypes. During the 72-h observation period, at least five different types of transcripts accumulation time profiles were identified in the *VvSTS* gene family in response to PV inoculation (Fig. 5 and Additional file 5: Figure S2). For each of the expression time profiles identified, its shape was almost identical among the nine grapevine genotypes, although the extent of the time-dependent changes was drastically different among them. The observed five time profiles are described in detail below.

A first type of time profile, exclusive of the only two genes belonging to the phylogenetic A group, namely *VvSTS1/2,* showed a remarkable and progressive increase after 16 and 24 hpi, followed by a flat or slightly decreasing trend thereafter (Fig. 5 and Additional file 5: Figure S2). Among the nine grapevine genotypes compared here, the highest levels of transcriptional activation of *VvSTS1/2* in response to the pathogen were detected for SOL and ISA hybrids (Fig. 5 and Additional file 2: Table S6), which, as pointed out above (Fig. 4), were the same genotypes which also showed the highest level of constitutive expression of the same genes. In the two hybrids, the PV-induced transcriptional activation of *VvSTS1/2* was approximately from three to four times higher at 24 hpi compared to 0 hpi, a difference that was maintained by the end of the observation period (72 hpi). For the SYL genotype, which also showed a comparatively high level of constitutive expression of *VvSTS1/2* (Fig. 4), the increase in the level of transcription at 24 hpi was less pronounced in comparison to SOL and ISA, with the consequence that the amount of transcripts detected in all leaf samples collected after infection was always significantly lower than that of the two hybrids (Fig. 5 and Additional file 2: Table S6). Among the subgroup of the *V. vinifera* varieties, the highest levels of induction of *VvSTS1/2* in response to PV was detected for ROS and ROM, for which the amount of transcripts at 24 hpi was approximately two times higher than that at 0 hpi. The level of transcription in all the leaf samples collected after infection in these two varieties was significantly higher than that in the remaining four *V. vinifera* varieties and substantially similar to that of the SYL accession. No or negligible time-dependent changes in the expression of *VvSTS1/2* were detected for the remaining components of the *V. vinifera* genotypes subgroup, namely CHA, ALE, CAN, and TRE (Fig. 5 and Additional file 2: Table S6).

A second type of time profile, exclusive of the C-group genes, namely *VvSTS22–24, VvSTS25/56, VvSTS27–30,* and *VvSTS31* (Fig. 5 and Additional file 5: Figure S2), showed a rapid and massive increase after 16 and 24 hpi, followed by a strong decreasing trend thereafter. Again (see the preceding time profile), the highest levels of induction in response to the pathogen was detected in the SOL and ISA hybrids (Fig. 5 and Additional file 2: Table S6). In these two genotypes, transcriptional activation at peak intensity (24 hpi) was approximately from four to eight times higher than at 0 hpi, a difference that decreased to two-three times by the end of the observation period (72 hpi). After the two hybrids, the third most responsive group of grapevine genotypes comprised again ROM, ROS and SYL, whose increase in the levels of transcription at 24 hpi was less pronounced than for SOL and ISA (1.5–2 times higher than that at 0 hpi). After 72 hpi, in these three genotypes, the differences in transcriptional activation respect to 0 hpi ranged from nihil to two times. No or negligible time-dependent changes in the expression of the aforementioned C-genes were detected in the remaining components of the *V. vinifera* subgroup, namely CHA, ALE, CAN, and TRE (Fig. 5 and Additional file 2: Table S6).

A third type of time profile was shown by the *VvSTS3/4*, *VvSTS5/6*, *VvSTS12*, *VvSTS13,* and *VvSTS14* genes, which, based on the high sequence identity of their protein products, cluster in a distinct subgroup within the B-group genes (Fig. 2). Such time profile showed a rapid and massive increase after 16 and 24 hpi, followed by a strong decrease at 48 hpi and, in certain grapevine genotypes, by a new increase at 72 hpi (Fig. 5 and Additional file 5: Figure S2). Once more (see above), the amount of transcripts detected at 24 hpi was significantly the highest in the two hybrids, and in particular in SOL (approximately ten times higher than at 0 hpi). In SOL and ISA, despite the decrease in the level of expression observed at 48 hpi, the amount of transcripts remained always significantly higher than that of the remaining genotypes and increased again significantly at 72 hpi (Fig. 5 and Additional file 2: Table S6). Among the six *V. vinifera* varieties the highest level of induction at 24 and 72 hpi was detected for ROS and ROM, which showed, in response to the pathogen, a similar temporal expression pattern among each other and similar in turn to that of SYL (Fig. 5 and Additional file 2: Table S6). Finally, it is worth noting that the remaining four *V. vinifera* varieties, namely CHA, ALE, CAN, and TRE, although showing a peak in transcripts levels at 24 hpi, failed to produce a new increase during the period 48–72 hpi, as all the remaining genotypes did (Fig. 5 and Additional file 2: Table S6).

A fourth type of time profile showed a linear and steady accumulation of transcripts starting from 16 to 24 hpi onwards. Such type of time profile was shown by the *VvSTS7/8*, *VvSTS9–11*, *VvSTS15*, *VvSTS16–18* and *VvSTS19* genes (Fig. 5 and Additional file 5: Figure S2), which, based on phylogenetic analysis, form another distinct subgroup within the B-group genes (Fig. 2). For this type of time profile, three sets of genotypes can be identified, based on transcripts accumulation in response to PV (Fig. 5 and Additional file 2: Table S6). The first set includes the two hybrids SOL and ISA, which showed the highest levels of transcripts accumulation during the progression of PV infection (16–72 hpi); it is worth noting in such respect that PV-induced transcriptional activation was invariably stronger in the former than in the latter hybrid. The second set includes the *V. vinifera* varieties ROM and ROS, and the SYL accession, showing an intermediate accumulation of transcripts; for each of the aforementioned genes, ROM and SYL showed significantly higher amounts of transcripts compared to ROS (Fig. 5 and Additional file 2: Table S6). The third set consists of the remaining four *V. vinifera* varieties, in which the level of induction of the aforementioned B-genes was very low or even null (Fig. 5 and Additional file 2: Table S6).

Finally, no significant expression change following PV-inoculation was observed for the two B-genes *VvSTS20* and *VvSTS21*, for which neither significant or biologically relevant differences were found among the nine grapevine genotypes (Fig. 5 and Additional file 2: Table S6).

No change in the temporal expression of *STS* genes was observed in the mock-inoculated plants (data not shown).

### Following *P. viticola* inoculation, the genes coding for the VvMYB14 and VvMYB15 transcription factors showed distinct time profiles of transcripts accumulation, each of which mirrored that of a distinct subset of the B-group stilbene synthase genes

**Fig. 6:**
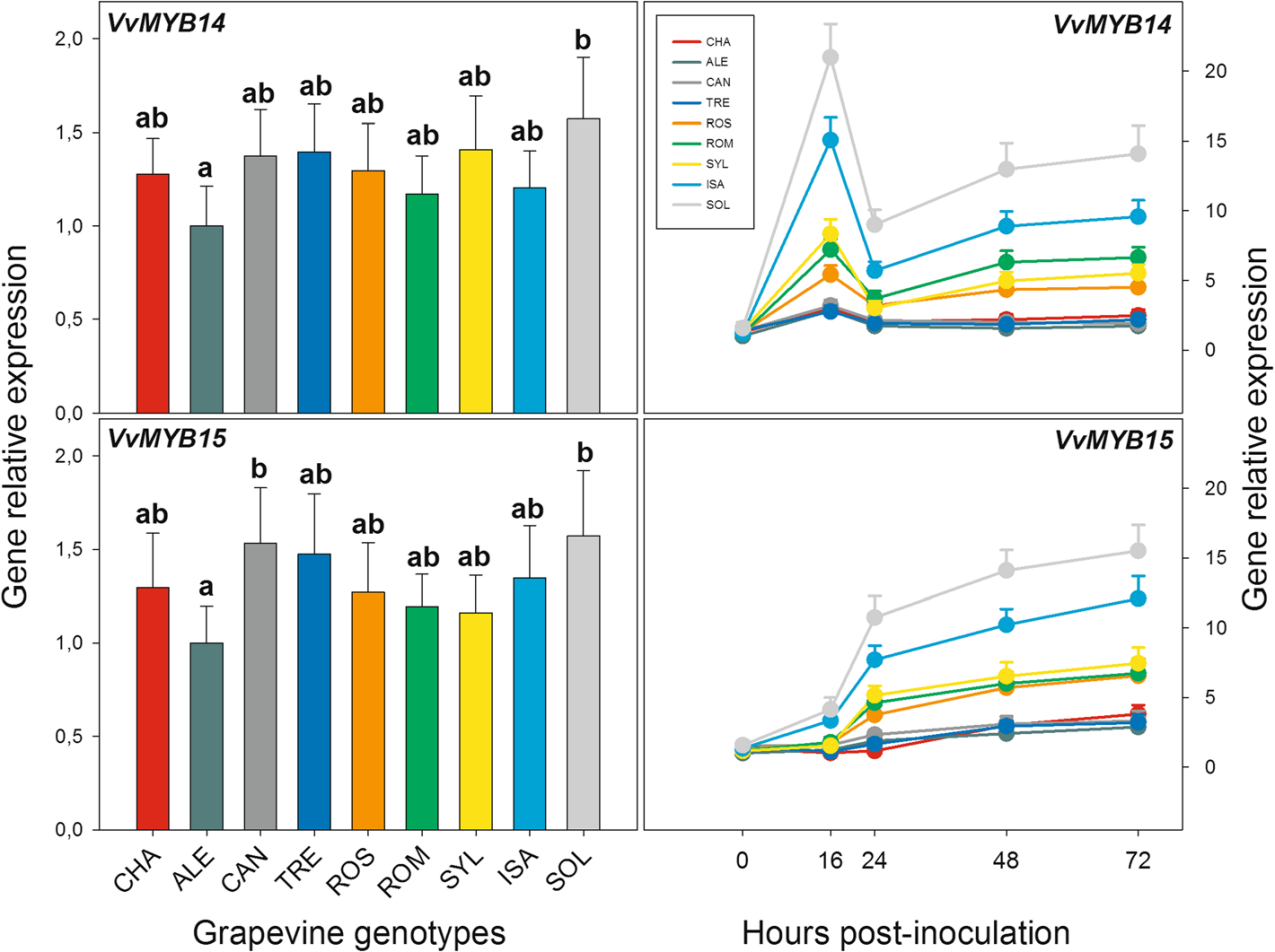
Relative gene expression levels of the genes coding for the two transcription factors *VvMYB14* and *VvMYB15* in the leaves of the nine grapevine genotypes of Fig. 1 collected either before inoculation with *Plasmopara viticola* (left panels) or up to 72 h after inoculation (right panels). For each gene, relative expression levels were calculated by setting a value of 1 for the lowest value among the nine genotypes in each of the five time points considered. Normalization and sample replication as in Fig. 3. For the left panels, different letters denote significant differences according to the Tukey’s test (*p* ≤ 0.01). In the right panels, statistical evaluation of the differences among the nine grapevine genotypes and among the five sampling times is reported in Additional file 3: Table S7. Grapevine genotypes: CHA = Chasselas, ALE = Aleatico, CAN=Canaiolo nero, TRE = Trebbiano toscano, ROS = Rossetto, ROM = Romanesco, SYL = accession of *V. vinifera* subsp. *sylvestris,* ISA = Isabella, SOL = Solaris

Figure 6 and Additional file 3: Table S7 present the constitutive expression levels (0 hpi) and the transcriptional time courses after PV inoculation of the genes coding for two transcription factors (TFs), namely VvMYB14 and VvMYB15*,* known to be involved in the transcriptional regulation of the *STS* genes [33]. The constitutive expression of both TFs immediately before PV-infection showed no significant or biologically relevant differences among the nine grapevine genotypes (Fig. 6, left panels). Following PV inoculation, distinct time profiles of transcripts accumulation were observed for the two TFs (Fig. 6, right panels). For each TF, the shape of the time profile was similar among the nine grapevine genotypes, although ample differences among them were noticed in terms of extent of transcriptional activation. When compared to the genes whose transcription are supposed to regulate, each TF mirrored one of the time profiles previously described for the B-group of the *VvSTS* genes. In particular, the *VvMYB14* time profile was similar in shape to that of the *VvSTS3/4*, *VvSTS5/6*, *VvSTS12*, *VvSTS13* and *VvSTS14* genes [compare Fig. 6, right panels, and Fig. 5; see description for the third time profile of the *VvSTS* genes in the preceding section of the Results], except that the peak of transcripts accumulation occurred earlier, i.e. at 16 hpi instead of at 24 hpi. Similar to the aforementioned B-*STS* genes, the initial accumulation of *VvMYB14* transcripts (16 hpi) was significantly higher in SOL and ISA (approximately 14 and 12 times higher than at 0 hpi, respectively), intermediate in the two *V. vinifera* varieties ROS and ROM, as well as and in the SYL accession (about from 5 to 7 times higher than at 0 hpi) and comparatively lower in the remaining four *V. vinifera* varieties, i.e. CHA, TRE, CAN and ALE (about 2–3 times higher than at 0 hpi; Fig. 6 and Additional file 3: Table S7). After the decrease at 24 hpi, which occurred in all genotypes, a subsequent new increase in transcripts amount was noticed, but only in SOL and ISA and, to a lesser extent, in SYL, ROS and ROM (Fig. 6).

The time expression profile of *VvMYB15* after PV inoculation was found to mirror that of the *VvSTS7/8*, *VvSTS9–11*, *VvSTS15*, *VvSTS16–18* and *VvSTS19* genes [compare Fig. 6, right panels, and Fig. 5; see the description for the fourth time profile of the *VvSTS* genes in the preceding section of the Results]. Such time profile was characterized by a steady accumulation of transcripts, starting from the first stages after PV inoculation (16–24 hpi) until 72 hpi. Similar to *VvMYB14* (see above)*,* examining the *VvMYB15* time profile allowed to identify three groups of genotypes, which can be distinguished among each other on the basis of the amount of the transcripts accumulated in response to PV. The first group included SOL and ISA hybrids, which showed the highest levels of accumulation of transcripts during the different stages of infection (12 and 15 times higher at 72 than at 0 hpi, respectively). The second group, represented by the *V. vinifera* varieties ROM and ROS and by the SYL accession, showed an intermediate accumulation of transcripts (approximately 6–7 times higher at 72 hpi than at 0 dpi). Finally, the third group included the remaining four *V. vinifera* varieties, namely CHA, TRE, CAN and ALE, in which the level of induction of *VvMYB15* in response to PV was very low, comparatively speaking, being approximately 2–3 times higher at 72 hpi than at 0 hpi (Fig. 6 and Additional file 3: Table S7).

No change in the temporal expression of *MYB* genes was observed in the mock-inoculated plants (data not shown).

### Following *P. viticola* inoculation, the chalcone synthase genes were up-regulated in the susceptible grapevine genotypes and down-regulated in the resistant ones

Of the five genes coding for CHS identified in the *V. vinifera* genome (see above), *VvCHS4* and *VvCHS5* were not considered further here, because the level of their transcripts was very low or even null in the plant material under study (data not shown).

**Fig. 7:**
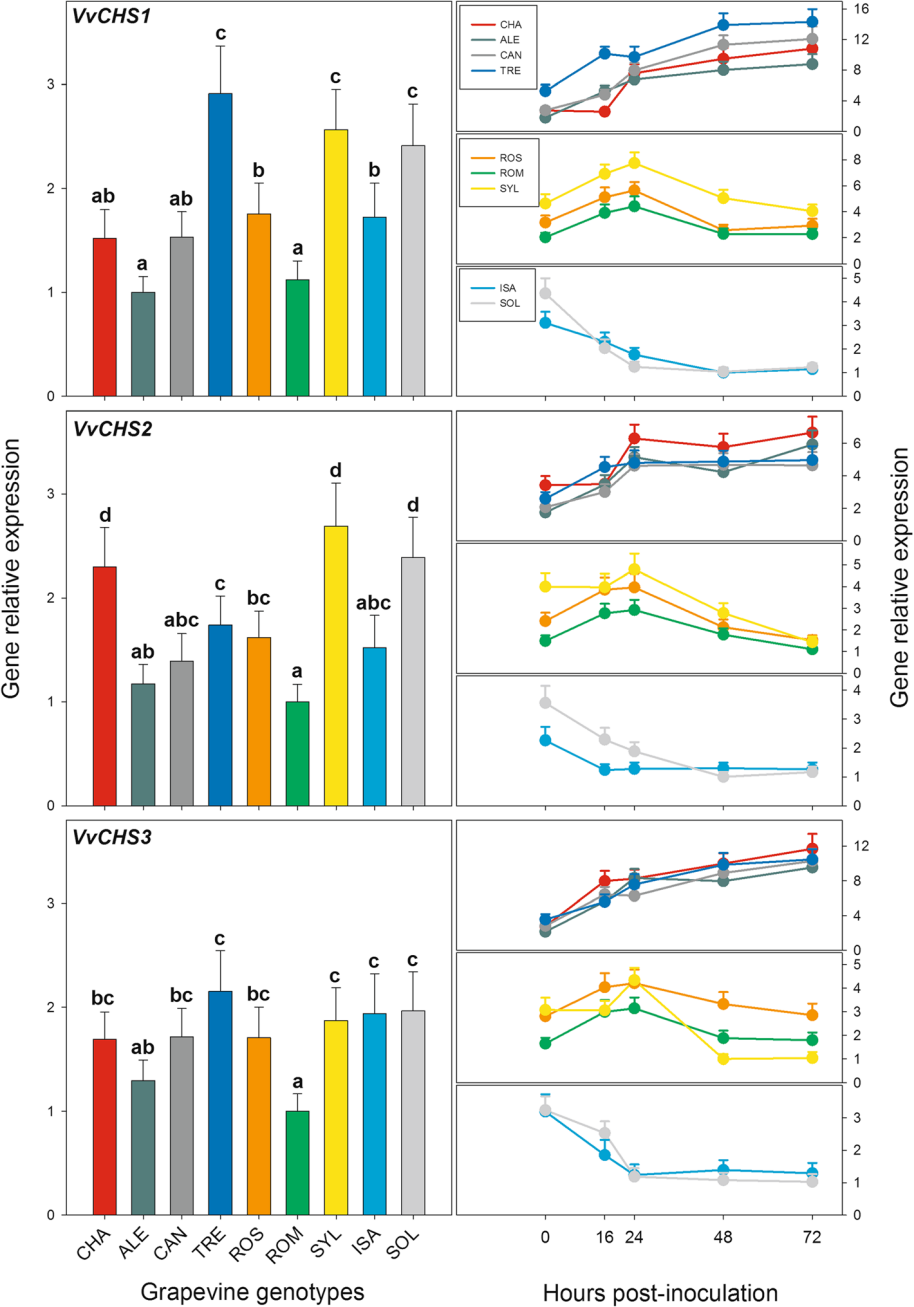
Relative expression levels of the *Vitis vinifera* chalcone synthase genes (*VvCHS*) in the leaves of the nine grapevine genotypes of Fig. 1 collected either before inoculation with *Plasmopara viticola* (left panels) or up to 72 h after inoculation (right panels). For each gene, relative expression levels were calculated by setting a value of 1 for the lowest value among the nine genotypes in each of the five time points considered. Normalization and sample replication as in Fig. 3. For the left panels, different letters denote significant differences according to the Tukey’s test (p ≤ 0.01). In the right panels, statistical evaluation of the differences among the nine grapevine genotypes and among the five sampling times is reported in Additional file 3: Table S8. Grapevine genotypes: CHA = Chasselas, ALE = Aleatico, CAN=Canaiolo nero, TRE = Trebbiano toscano, ROS = Rossetto, ROM = Romanesco, SYL = accession of *V. vinifera* subsp. *sylvestris,* ISA = Isabella, SOL = Solaris

Figure 7 and Additional file 3: Table S8 present the constitutive expression levels (0 hpi) and the transcriptional time courses after PV inoculation of *VvCHS1*, *VvCHS2*, and *VvCHS3* genes. Significant differences were found in the constitutive expression of *VvCHS* genes among the grapevine genotypes considered (Fig. 7, left panels), which, however, and contrary to the *VvSTS* genes of the A and C groups (see Figs. 4 and 1), did not appear to be related to the different proneness to develop downy mildew symptoms upon PV infection. In fact, in the case of *VvCHS1* the amount of transcripts was significantly higher in TRE, SOL and SYL, compared to the other genotypes considered, while, for *VvCHS2*, the highest transcription levels were detected in SOL, CHA and SYL. Finally, as regards *VvCHS3*, the differences found among the different genotypes before PV inoculation were less pronounced than for the other two *VvCHS* genes, even if the quantity of its transcripts was higher in SOL, SYL and TRE, compared to the other genotypes considered.

Following PV inoculation, three types of time profiles were observed in all the *VvCHS* genes (Fig. 7, right panels), allowing to distinguish as many groups of grapevine genotypes. In the highly susceptible *V. vinifera* cultivars TRE, CAN, ALE and CHA, the transcription levels of all the *VvCHS* genes, irrespective of the constitutive differences at 0 hpi, increased steadily and significantly from the initial stages of infection (16–24 hpi) onwards, reaching their maximum at 72 hpi (Fig. 7, right panels and Additional file 3: Table S8). In the time profiles of the moderately susceptible grapevine genotypes, namely SYL, ROM and ROS, instead, a general and significant increase in the transcription levels occurred until 24 hpi, followed by a rapid decrease in the amount of transcripts in the next 48 h (Fig. 7, right panels and Additional file 3: Table S8). In the SOL and ISA hybrids, finally, PV inoculation led to a steady and significant reduction of transcripts accumulation starting from the early stages of infection (16–24 hpi), indicating that in the two downy mildew-resistant genotypes the transcription of the *VvCHS* genes was rapidly and almost completely suppressed in response to the pathogen (Fig. 7, right panels and Additional file 3: Table S8).

No change in the temporal expression of *CHS* genes was observed in the mock-inoculated plants (data not shown).

## Discussion

### Constitutive and/or pathogen-induced resistance traits to downy mildew can be found within both cultivated and wild *V. vinifera* germplasm

Although the *V. vinifera* germplasm is considered to be highly susceptible to downy mildew [36], previous studies indicated that intraspecific differences in susceptibility do exist [11–17], thus supporting the view that constitutive and/or induced defense mechanisms could be differentially effective among *V. vinifera* varieties in limiting disease progression [37, 38]. The results reported here show that proneness to undergo PV infection varies substantially also among *V. vinifera* varieties traditionally cultivated in Italy, being ROM and ROS remarkably less susceptible than ALE, CAN, and TRE. Previous work of ours on the same plant material [5, 16] suggested that leaf morphological traits and stilbene production might be involved in limiting downy mildew infection in the less susceptible varieties. As suggested by the results obtained for the SYL wild accession, adaptive resistance traits to PV could also be found outside of the cultivated *V. vinifera* germplasm. In such context, a recent analysis carried out on an extensive collection of *V. vinifera* subsp. *sylvestris* accessions representing the genetic variability of the European wild grape still present in Germany, revealed that many genotypes show good tolerance against several grapevine diseases, including downy- and powdery mildew (*Erysiphe necator*), which were both introduced only 150 years ago from North America [19, 39].

### Elevated constitutive levels of stilbene synthase genes and their vigorous transcriptional activation upon *P. viticola* infection may contribute to basal immunity against the downy mildew of grapevine

Plant immunity is made up of two levels: an evolutionarily ancient basal immunity is complemented by a more efficient and specific second line of defence, which often evolves as a result of a long arms race between the pathogen and the host plant [39]. Since cultivated grapevine (*V. vinifera* subsp. *vinifera*) and its wild ancestor (*V. vinifera* subsp. *sylvestris*) did not evolve together with PV, they represent harmless hosts and, in contrast to North American wild species of *Vitis*, lack the efficient second layer of innate immunity against downy mildew. The fact that certain genotypes of *V. vinifera* in this study showed lower level of susceptibility to PV is likely to be due to a more efficient basal immunity.

Keeping in mind that stilbenes, as important phytoalexins, represent a central element of the basal immunity in grapevine, then the observed differences both in the constitutive expression of *VvSTS* genes, and in their levels of induction in response to PV, as related to the variable incidence and severity of disease symptoms exhibited by the nine grapevine genotypes studied here, clearly suggest that stilbene biosynthesis may be crucial to successful defence against the pathogen.

Constitutive resistance to grapevine diseases, among which downy mildew, has been linked to high constitutive levels of antimicrobial compounds, such as inositol, caffeic acid and stilbenes, in uninfected leaves of resistant wild species [40, 41]. Constitutive resistance was also suggested by the high levels of expression of genes related to stress and defence, including *STS* genes, in resistant grapevines, as compared to the lower expression levels observed in susceptible uninoculated grapevines [40–44]. The comparatively high constitutive *VvSTS* expression observed here in the leaves of the resistant and moderately susceptible grapevines, especially in the case of the A and C genes, could therefore have a predictive value about the capacity of a particular genotype to early interrupt or limit a subsequent infection brought about by *P. viticola*.

Our results also indicate that, for almost all the functional *VvSTS* genes and in all the grapevine genotypes, the extent of transcriptional activation in the first 72 h following PV inoculation is inversely related to the degree of susceptibility, and hence can reflect and pre-empt the extent of symptoms development. As far as the B-group of *STS* genes is concerned, our results confirm the previous findings of Vannozzi et al. [26], who reported that the increase in the biosynthetic capacity of the stilbene pathway in response to different biotic and abiotic stresses, including the downy mildew disease, can be contributed mainly by the B group of the *VvSTS* family members.

The dissection made here among the group-specific temporal expression patterns within the *VvSTS* genes family in response to PV inoculation might be of help in the interpretation of previous results obtained from studying *VvSTS* expression as a whole (i.e. by using Northern blot assays or PCR with generic and probably highly conserved primers) in response to biotic or abiotic stresses [45–49]. In such studies, in fact, the timing of the *VvSTS* total transcripts accumulation in response to different stresses was often observed to be biphasic in shape, and this led some authors to hypothesize that the *VvSTS* gene family members might be divided into two groups, one of which expressed early, with a rapid degradation of mRNA, and the other expressed later on, with a more stable mRNA [45].

### Genome-wide analysis of the stilbene synthase gene family in grapevine reveals that the infection brought by *P. viticola* elicits a co-ordinated and sequential transcriptional activation of distinct gene subsets, each of which may be regulated by a distinct and specific MYB transcription factor

Our results suggest that the *VvSTS* genes within the A and C groups might be responding to transcriptional signals whose nature and/or timing clearly differ from those activating the B genes. In turn, we show here that even within the same B-group genes, two clearly distinguishable transcriptional time profiles can be identified following PV inoculation, indicating the involvement of distinct mechanisms/timing of transcriptional regulation.

Several lines of evidence in recent years indicated that two R2R3-type V-myb myeloblastosis viral oncogene homolog (MYB) TFs, designated as VvMYB14 and VvMYB15, are involved in the transcriptional regulation of stilbene biosynthesis in grapevine [33, 48–50]. These TFs strictly co-express with certain *VvSTS* genes both in the leaf tissues under biotic and abiotic stresses (UV-C irradiation, wounding, and downy mildew infection) and in the skin and seeds of healthy developing berries during maturation [33, 50] . Because we found no difference among the nine grapevine genotypes in the constitutive expression of both *VvMYB14 and VvMYB15,* the differences we found instead in the constitutive expression of *VvSTS* genes, cannot be explained by the activity of the two aforementioned TFs. In other words, the comparatively higher constitutive expression of *VvSTS* genes detected in certain grapevine genotypes might be regulated by TFs other than VvMYB14 and VvMYB15. Indeed, a systems-orientated analysis recently performed by Wong et al. [50] indicated a role for the *VvMYB13* gene, which is the still uncharacterized closest homologue of *VvMYB15*, in regulating stilbene biosynthesis in leaves under non-stressed conditions. It has been also suggested that other TFs, such as WRKYs, NAC, and ARF2, which co-express with certain *VvSTS* genes or are activated in response to stilbene-inducing hormones, might be involved in the regulation of stilbene biosynthesis [48, 51]. Clearly, further studies are needed to ascertain whether VvMYB13 and/or other TFs regulating stilbene biosynthesis in vegetative tissues under non-stressed conditions, could be responsible for the differences in the constitutive expression of *VvSTS* genes in the considered genotypes.

On the other hand, our results strongly suggest the involvement of VvMYB14 and VvMYB15 in regulating of the expression of the B-group *VvSTS* genes in response to PV, thus confirming the findings of Holl et al. [33], but adding that each of the aforementioned TFs might specifically act on a distinct subset of the B genes. It is also worth noting that, following PV inoculation, the transcriptional activation of *VvMYB14,* but not that of *VvMYB15,* preceded (16 hpi) the initial induction of its putative B-genes targets (24 hpi).

Taken together, the above results suggest that PV inoculation elicits in the nine grapevine genotypes a co-ordinated and sequential transcriptional activation of distinct subsets of *VvSTS* genes, whose extent is inversely proportional to the observed proneness, high, moderate or low, depending on the genotype considered, to develop downy mildew symptoms once infection becomes established. Both the timing and the extent of the distinct patterns of *VvSTS* overexpression in response to the pathogen might be orchestrated by distinct TFs, among which VvMYB14 and VvMYB15, whose activation in response to PV seems to be strictly concomitant with that of their respective *STS* targets, with which they are supposed to interact in a highly specific manner.

The observed differences in the *VvMYBs* expression patterns in response to PV prompt to ask about the stress signal(s) acting upstream of their own transcription. In this respect, jasmonic acid-, salicylic acid- and ethylene-mediated signalling pathways have been proposed to come into play [51–55]. Indeed, Duan et al. [49] demonstrated that *STS* inducibility among *V. sylvestris* genotypes correlated mainly with the differences in *VvMYB14* transcripts accumulation, and showed that jasmonic acid-mediated signalling, but not the salicylate-mediated one, were involved in the *VvMYB14* transcriptional activation. However, the precise mechanisms of the hormone-mediated regulation of stilbene biosynthesis, as well as the TFs involved, remain to be defined.

### During the early stages of *P. viticola* infection, an antagonistic interaction between flavonol and stilbene biosynthesis arises, whose outcome may determine the subsequent incidence and severity of the downy mildew symptoms

Calcone synthases (CHSs), the key enzymes responsible for the biosynthesis of chalcones and their derivatives (flavonols, proanthocyanidins and anthocyanins) share a high degree of structural and functional homology with STSs, as illustrated by the fact that both enzymes compete for the same substrates [56]. For each of the three *VvCHS* genes analysed here, we found significant differences in the levels of constitutive expression among the nine genotypes considered. However, and contrary to *VvSTSs*, such differences in constitutive expression were not related to different levels of resistance/susceptibility to PV. Moreover, and again contrary to *VvSTS*, remarkable differences among the nine grapevine genotypes were found, not only in terms of level but also in terms of temporal pattern, concerning *VvCHS* transcripts accumulation after PV inoculation. Looking for similarities and differences, three clearly distinguishable *VvCHS* time profiles were identified, each of which representing a group of grapevine genotypes. In particular, the three genotypes groups so identified closely reflected the high-moderate-low PV susceptibility groups already identified based on the incidence and severity of disease symptoms (see above). However, in an opposite specular manner respect to *VvSTS*, PV inoculation elicited a remarkable and sustained overexpression of all the three *VvCHS* genes in those genotypes found to be the most susceptible to downy mildew, whereas in the most resistant ones, on the contrary, *VvCHSs* were even down-regulated.

Clearly, the results reported here suggest the existence of an interaction between the metabolic pathways leading to the synthesis of stilbenes and flavonoids, whose outcome affects disease incidence and severity. Evidence for the existence of a crosstalk between these two pathways in grapevine cells came out from gene expression analysis in different tissues at various developmental stages of the *V. vinifera* cultivar Corvina, carried out by Vannozzi et al. [26]. Tissues in which *STSs* expression levels were generally low, such as stem, bud, young leaves, rachis at fruit set and developing berries, showed high constitutive expression of *CHS* genes. Conversely, the expression of *CHS* genes was suppressed in tissues in which the *STS* genes were actively transcribed, i.e. roots, senescing leaves, maturing rachis and withering berries. Most importantly, these same authors reported that, aside from constitutive expression, a similar picture of reciprocal antagonism among the *VvSTS* and the *VvCHS* gene families emerged when grape leaves were challenged with UV-C exposure or PV inoculation. Both stress treatments, in fact, resulted in dramatic increase in *VvSTS* transcription, whereas the expression of *VvCHS* genes was strongly suppressed [26]. Coherently, our results suggest for the first time that an antagonistic relationship might exist between flavonol biosynthesis and stilbene biosynthesis in an array of grapevine genotypes covering the whole range of susceptibility to downy mildew. The induction of *VvCHS* genes in response to PV, observed in the most susceptible *V. vinifera* varieties, and the consequent probable accumulation of flavonoids in the early stages of infection, could hinder the activation of effective defence mechanisms, thus predisposing the plant to develop disease symptoms later on. In contrast, the total suppression of *CHS* transcription in resistant genotypes could favour the induction of *VvSTS* genes and the consequent accumulation of stilbenes, which are known to play an important role in defence responses against several pathogenic fungi, including downy mildew [25, 27]. To further support the above concept, in the less susceptible *V. vinifera* genotypes, namely ROM, ROS and SYL, laying in the middle between the highly susceptible and the resistant grapevines, PV inoculation seemed to elicit an early activation of the flavonoid pathway, which however was rapidly shut down thereafter, so that the phenylpropanoid pathway could be conceivably diverted towards stilbenes biosynthesis.

## Conclusions

In the present work, the expression of each of the members of the grapevine stilbene synthase gene family was analysed immediately before and up to 72 h after artificial inoculation with *P. viticola*, the causal agent of the downy mildew disease. As the plant material, we used a group of grapevine accessions comprising both cultivated and wild *V. vinifera* genotypes, expected to be inherently susceptible, as well as hybrids between *V. vinifera* and American *Vitis* species, expected to be inherently resistant. Phytopathological evaluation run 7 days after pathogen inoculation showed that, within the compared grapevine accessions, the incidence and the severity of downy mildew symptoms ranged from high to nihil, being low to moderate in a subgroup of *V. vinifera* genotypes.

In all the grapevine genotypes examined, the constitutive expression as well as the extent of transcriptional activation of the stilbene synthases genes soon after pathogen inoculation were found to be inversely proportional to the incidence and severity of disease symptoms. The infection brought by *P. viticola* appeared to elicit a co-ordinated and sequential transcriptional activation of distinct stilbene synthase genes subsets, each of which may be regulated by a distinct and specific MYB transcription factor. Taken together, the results reported here suggest that the induction of stilbene biosynthesis may contribute to the basal immunity against the downy mildew of grapevine and therefore it could be an adaptive resistance trait to look for in both cultivated and wild *V. vinifera* germplasm.

An alongside analysis of the temporal expression patterns of the chalcone synthase genes suggested that during the early stages of *P. viticola* infection an antagonistic interaction between flavonol and stilbene biosynthesis might occur, whose outcome might determine the subsequent incidence and severity of the downy mildew symptoms. Further detailed studies are needed to confirm the existence of the antagonistic crosstalk between the metabolic pathways that lead to the synthesis of stilbenes and flavonoids in resistant and susceptible genotypes in response to *P. viticola*, as well as to decipher the possible regulatory mechanisms involved.

## Methods

### Plant material

Five *V. vinifera* subsp. *vinifera* varieties, some of which contribute to the production of protected designation of origin (PDO) and protected geographical indication (PGI) wines in Central Italy, were used in the present study: black berried “Aleatico” (ALE) and “Canaiolo nero” (CAN), and white berried “Romanesco”, “Trebbiano toscano” (TRE) and “Trebbiano giallo”, here referred to by using its official synonym, i.e. “Rossetto” (ROS). Previous ampelographic description and DNA analysis confirmed the correct identification of the five varieties [57]. The five grapevines were selected because of their different level of resistance to downy mildew previously observed both in field and in controlled environment [5, 16]. Furthermore, a *V. vinifera* subsp. *sylvestris* accession (SYL) collected in Monti Cimini area (Viterbo, Italy) was included in the present study because of the increasing interest for this wild ancestor of cultivated grapevine as a source of useful traits to be introduced into the subsp. *vinifera* [19, 20, 39]. The downy mildew-susceptible *V. vinifera* variety “Chasselas” (CHA), the first-generation hybrid “Isabella” (ISA) (*V. labrusca x V. vinifera*), and the interspecific variety “Solaris” (SOL) [‘Merzling’ × (‘Saperavi severneyi’ x ‘Muscat Ottonel’)], resulting from multiple backcrossing among American species, *V. amurensis* and *V. vinifera* [58], both of which known to be resistant to PV, were included in the present study as “references” genotypes, to verify the effectiveness of the artificial PV infection.

### Evaluation of the degree of resistance to *P. viticola* under controlled conditions

Two year-old potted plants of each grapevine genotype obtained from woody cuttings were used for the PV inoculation test under controlled conditions. The plants, each with three shoots, were grown in 6.5 L pots filled with a 2:1 mixture of commercial peat and pumice in a greenhouse at 22 ± 2 °C and 75 ± 5% relative air humidity, under a 16 h photoperiod whit natural daylight supplemented with high pressure metal halide lamps (OSRAM, Germany). The supplemental system was applied when photosyntetically active radiation dropped below a photosynthetic flux density of 900 μmol m^− 2^ s^− 1^. During growth, the plants were regularly watered.

At the ten developed leaf stage, fifteen plants for each genotype were inoculated by spraying an aqueous suspension of 1 × 10^5^ PV zoosporangia mL^− 1^ onto the abaxial leaf surface. The inoculum was obtained from naturally infected plants showing downy mildew symptoms and maintained on the susceptible grape variety ‘Malvasia del Lazio’. Fifteen mock-inoculated plants for each genotype were obtained by spraying the leaves with distilled water only. Then, the mock-inoculated plants were kept isolated from the PV-inoculated ones and grown under identical environmental conditions.

The degree of resistance to PV of the nine grapevine genotypes was evaluated 7 days after inoculation (days post inoculation, dpi). For each grapevine genotype, all the PV inoculated plants were used for measuring both disease incidence, which was expressed as the percent of symptomatic leaves over the total leaves, and disease severity, i.e. the percent of symptomatic leaf surface over the total leaf surface, which has been calculated according to the method proposed by Cadle-Davidson [13]. The statistical significance of the differences observed between the means of the two parameters in the nine genotypes was evaluated by one-way ANOVA, followed by the Tukey’s test (*p* ≤ 0.05). Percentages were transformed to arcsine-square root values before analysis.

### Database analyses and identification of target and reference genes

The sequences of the genes of interest were obtained from the NCBI (National Center for Biotechnology Information) Reference Sequence database, which provides, for each species whose genome has been sequenced, a comprehensive, non-redundant, well-annotated set of sequences (RefSeq), including genomic DNA, transcripts, and proteins. The Reference Sequences of genes coding for STS and CHS synthases were recognized using two different strategies. First, the available grapevine STS and CHS cDNA sequences [26, 32, 59] were utilized in the NCBI Reference Sequence database for BLAST search. A further search was carried using the words ‘stilbene synthase’ and ‘chalcone synthase’ as query terms in in the *V. vinifera* genome view page.

The Reference Sequences of the two transcription factors VvMYB14 and VvMYB15 were identified by using the known sequences of the genes corresponding to the identifiers VIT_07s0005g03340 and VIT_05s0049g01020 [33] on the 12X V1 genome assembly of the PN40024 genotype.

The respective NCBI mRNA and RefSeq protein sequences were retrieved for each identified gene (Additional file 1: Tables S1, S3 and S4). The identity of the recognized sequences was confirmed by testing the respective RefSeq protein sequences in the Interpro, Simple Modular Architecture Research Tool (SMART), Pfam hidden Markov models (HMMs), and Conserved Domains Database (CDD) databases. The RefSeq mRNA sequences were used as templates to identify specific primers for expression analysis (Additional file 1: Tables S2-S4).

Initially, seven candidate genes displaying stable expression in distinct grape tissues under different stress conditions [60–63] were chosen to recognize the most appropriate reference gene/genes. The identified candidate reference genes encoded the following proteins: EF1-α elongation factor (*VvEF1α*), SAND protein family (*VvSAND*), glyceraldehyde-3-phosphate dehydrogenase (*VvGAPDH*), 60S ribosomal protein L18 (*Vv60SRP*), Actin7 (*VvACT7*), ubiquinol-cytochrome *c* reductase complex chaperone (*VvUQCC*), and V-type proton ATPase 16 kDa proteolipid subunit (*VvVATP16*) (Additional file 1: Table S5). The corresponding mRNA RefSeq were used as templates to design specific primers for the analysis of their expression (Additional file 1: Table S5).

### Phylogenetic analysis

A multiple sequence alignment of the identified VvSTS deduced proteins (Additional file 1: Table S1) was performed by ClustalX version 1.83 [64], using the Gonnet series as protein weight matrix and parameters set to 10 gap open penalty, 0.2 gap extension penalty, negative matrix on and divergent sequences delay at 30%. Three CHS proteins corresponding to VvCHS1 (XP_002264019.1), VvCHS2 (NP_001267879.1) and VvCHS3 (NP_001268064.1) (Additional file 1: Table S3) were also included in the analysis. An unrooted phylogenetic tree was generated with the Neighbor-Joining method [65] using MEGA7 software [66]. The evolutionary distances, measured in terms of number of amino acid substitutions per site, were computed using the JTT matrix-based method. The rate variation among sites was modeled with a gamma distribution (shape parameter = 1). The reliability of the obtained tree was tested using bootstrapping with 1000 replicates.

### Gene expression analysis

Three uniform and apparently healthy leaves belonging to the 5th or the 6th node from the apex were sampled from both inoculated and mock-inoculated individual plants after 0, 16, 24, 48 and 72 h from PV inoculation. At each sampling time, a different and previously untouched individual plant was used. After excision, the leaves were immediately frozen in liquid nitrogen, and then stored at − 80 °C until RNA extraction. Total RNA extraction and cDNA synthesis were performed according to Paolacci et al. [67].

Quantitative RT-PCR analyses were performed as described in Paolacci et al. [67] and included three biological replicates, which derived from three distinct RNA extractions, RT and qRT-PCR reactions from three separate plants for each genotype at the five different points considered; in addition, for each biological replicate, three technical replicates were evaluated.

Specific primer pairs were designed for both target and reference genes (Additional file 1: Tables S2-S5) using the Beacon Designer 6 software (Stratagene, La Jolla, CA) as described in Paolacci et al. [67]. Only primer pairs generating a sharp peak by melting curve analysis (without unspecific products or primer-dimer artifacts) and showing efficiencies between 90 and 110% and R^2^ values (coefficient of determination) calculated for standard curves higher than 0.995 were selected for expression analysis of the target and references genes.

Raw C_t_ values were transformed to relative quantities using the delta-C_t_ formula, Q = E^ΔCt^, where E is the efficiency of the primer pair used in the amplification of a specific gene (100% = 2) and ΔC_t_ is the difference between the sample with the lowest C_t_ from the dataset and the C_t_ value of the sample in question. Only in the case of the comparison of the relative expression levels of selected members of the three phylogenetic groups of *VvSTS* gene family in the leaves of the nine grapevine genotypes collected before inoculation with *P. viticola* (Fig. 3), the formula used to convert C_t_ values into relative quantity was Q = 2^ΔCt^. This assumption was justified by the fact that the amplification efficiencies of the considered genes were approximately the same, ranging from 96 to 100%.

The expression stability of the seven candidate reference genes (Additional file 1: Table S5) was evaluated by the software program NormFinder [68], as described in Paolacci et al. [67]. The best combination of two genes proposed by NormFinder was that of VvEF1α and VvSAND, with a stability score considerably lower than that of the most stable gene (VvEF1α) taken alone, suggesting a more accurate normalization than that performed using the single most stable gene. The expression data of the genes of interest were, therefore, normalized using the geometric mean of the two reference genes VvEF1α and VvSAND and their normalized relative values provided as mean value ±SD. SDs on normalized expression levels were calculated according to the geNorm user manual (geNorm manual, update 8 July 2008). The statistical significance of the differences for the same genotype in the different five time points and among the different genotypes at a particular time point were evaluated by one-way ANOVA followed by Tukey’s test (*p* ≤ 0.01).

## Supplementary information

Additional file 1: **Table S1.** *Vitis vinifera* gene sequences coding for stilbene synthase (*VvSTS*) identified in the National Center for Biotechnology Information (NCBI) database. **Table S2.** List of primer pairs used in the present study for expression analysis of the *Vitis vinifera* stilbene synthase (*VvSTS*) genes. **Table S3.** *Vitis vinifera* gene sequences coding for chalcone synthase (*VvCHS*) identified in the NCBI database and used for the synthesis of oligonucleotides for expression analysis. **Table S4.** *Vitis vinifera* gene sequences coding for VvMYB14 and VvMYB15 transcription factors that specifically interact with the stilbene synthase promoter, identified in the NCBI database and used in the synthesis of oligonucleotides for expression analysis. **Table S5.** *Vitis vinifera* gene sequences coding for candidate reference genes identified in the NCBI database and used for the synthesis of oligonucleotides for the normalization of expression data in qRT-PCR analyses. (DOCX 30 kb)

Additional file 2: **Table S6.** Statistical evaluation of the differences (one-way ANOVA followed by Tukey test) among the relative expression levels of stilbene synthase (*VvSTS*) genes in the leaves of nine different grapevine genotypes (Gen, for genotypes acronyms, see the main text) after 0, 16, 24, 48, and 72 h from inoculation (hours post-inoculation, HPI) with *Plasmopara viticola*. For each of the stilbene synthase gene, the upper panel shows the statistical differences of the mean ± SD among the five sampling times for each grapevine genotype; the lower panel shows the statistical differences of the mean ± SD among the nine grapevine genotypes at each sampling time. Different letters denote statistically significant differences at *p* < 0.01; n.s., not statistically significant at the chosen probability threshold. (DOCX 59 kb)

Additional file 3: **Table S7.** Statistical evaluation of the differences (one-way ANOVA followed by Tukey test) among the relative expression levels of MYB transcription factors genes (*VvMYB14* and *VvMYB15*) in the leaves of nine different *Vitis vinifera* genotypes (Gen, for genotypes acronyms, see the text) after 0, 16, 24, 48, and 72 h from inoculation (hours post-inoculation, HPI) with *Plasmopara viticola*. For each of the MYB transcription factors genes, the upper panel shows the statistical differences of the mean ± SD among the five sampling times for each grapevine genotype; the lower panel shows the statistical differences of the mean ± SD among the nine grapevine genotypes at each sampling time. Different letters denote statistically significant differences at *p* < 0.01; n.s., not statistically significant at the chosen probability threshold. **Table S8.** Statistical evaluation of the differences (one-way ANOVA followed by Tukey test) among the relative expression levels of chalcone synthase genes (*VvCHS1–3*) in the leaves of nine different grapevine genotypes (Gen, for genotypes acronyms, see the text) after 0, 16, 24, 48, and 72 h from inoculation (hours post-inoculation, HPI) with *Plasmopara viticola*. For each of the chalcone synthase gene, the upper panel shows the statistical differences of the mean ± SD among the five sampling times for each grapevine genotype; the lower panel shows the statistical differences of the mean ± SD among the nine grapevine genotypes at each sampling time. Different letters denote statistically significant differences at *p* < 0.01; n.s., not statistically significant at the chosen probability threshold. (DOCX 28 kb)

Additional file 4: **Figure S1.** Leaves of the grapevine hybrid Solaris showing hypersensitive reaction seven days after inoculation with *Plasmopara viticola. (TIFF 2066 kb)*

Additional file 5: **Figure S2.** Heat maps of the relative expression levels of the whole set of the *Vitis vinifera* stilbene synthase genes (*VvSTS*) in the leaves of the nine grapevine genotypes of Fig. 1 collected up to 72 h after inoculation with *Plasmopara viticola*. The letter denoting the phylogenetic group to which each gene belongs is given in parentheses. For each gene, relative expression levels were calculated by setting a value of 1 for the lowest value among the nine genotypes in each of the five time points considered. Normalization and sample replication as in Fig. 3. For each gene, the differences in the relative expression levels were shown in color according to the scale and statistical evaluation of the differences among the nine grapevine genotypes and among the five sampling times is reported in Table S6. (ZIP 3113 kb)

## Data Availability

All data generated or analysed during this study are included in this published article and its supplementary information files.

## Abbreviations

CHS: Chalcone synthase
NCBI: National Center for Biotechnology Information
PV: *Plasmopara viticola*
SD: Standard deviation
STS: Stilbene synthase
TF: Transcription factor
