# Supplementary material for: Transcriptional regulation of stilbene synthases in grapevine germplasm differentially susceptible to downy mildew
Source: BMC Plant Biol. 2019 Sep 14;19:404. doi: 10.1186/s12870-019-2014-5 (PMC6744718; doi:10.1186/s12870-019-2014-5)
Supplement: Supplementary file 1 — Table S1. Vitis vinifera gene sequences coding for stilbene synthase (VvSTS) identified in the National Center for Biotechnology Information (NCBI) database. Table S2. List of primer pairs used in the present study for expression analysis of the Vitis vinifera stilbene synthase (VvSTS) genes. Table S3. Vitis vinifera gene sequences coding for chalcone synthase (VvCHS) identified in the NCBI database and used for the synthesis of oligonucleotides for expression analysis. Table S4. Vitis vinifera gene sequences coding for VvMYB14 and VvMYB15 transcription factors that specifically interact with the stilbene synthase promoter, identified in the NCBI database and used in the synthesis of oligonucleotides for expression analysis. Table S5. Vitis vinifera gene sequences coding for candidate reference genes identified in the NCBI database and used for the synthesis of oligonucleotides for the normalization of expression data in qRT-PCR analyses. (DOCX 30 kb) [file 12870_2019_2014_MOESM1_ESM.docx]

Manuscript title:

Transcriptional regulation of stilbene synthases in grapevine germplasm differentially susceptible to downy mildew.

Authors: Mario Ciaffi, Anna Rita Paolacci, Marco Paolocci, Enrica Alicandri, Valentina Bigini, Maurizio Badiani and Massimo Muganu.

The following Supplementary Tables are available for the aforementioned manuscript in the present additional file 1:

**Table S1.** *Vitis vinifera* gene sequences coding for stilbene synthase (*VvSTS*) identified in the National Center for Biotechnology Information (NCBI) database.

**Table S2.** List of primer pairs used in the present study for expression analysis of the *Vitis vinifera* stilbene synthase (*VvSTS*) genes.

**Table S3.** *Vitis vinifera* gene sequences coding for chalcone synthase (*VvCHS*) identified in the NCBI database and used for the synthesis of oligonucleotides for expression analysis.

**Table S4.** *Vitis vinifera* gene sequences coding for VvMYB14 and VvMYB15 transcription factors that specifically interact with the stilbene synthase promoter, identified in the NCBI database and used in the synthesis of oligonucleotides for expression analysis.

**Table S5.** *Vitis vinifera* gene sequences coding for candidate reference genes identified in the NCBI database and used for the synthesis of oligonucleotides for the normalization of expression data in qRT-PCR analyses.

**Table S1.** *Vitis vinifera* gene sequences coding for stilbene synthase (*VvSTS*) identified in the National Center for Biotechnology Information (NCBI) database.

| **Nomenclature**  **adopted** | **NCBI Reference Sequence**  **(mRNA)** | **NCBI Reference Sequence**  **(protein)** | **ORF (bp)** | **Nomenclature after**  **Vannozzi et al (2012)*** | **Chromosome**  **location** |
| --- | --- | --- | --- | --- | --- |
| *VvSTS1* | XM_002272093.4 | XP_002272129.1 | 1179 | *VvSTS6* | 10 |
| *VvSTS2* | XM_002271335.4 | XP_002271371.1 | 1179 | *VvSTS5* | 10 |
| *VvSTS3* | XM_010664197.2 | XP_010662499.1 | 1179 | *VvSTS41* | 16 |
| *VvSTS4* | XM_003634015.3 | XP_003634063.1 | 1179 | *VvSTS45* | 16 |
| *VvSTS5* | NM_001281005.1 | NP_001267934.1 | 1179 | *VvSTS48* | 16 |
| *VvSTS6* | XM_003634018.3 | XP_003634066.1 | 1179 | *VvSTS35* | 16 |
| *VvSTS7* | XM_002263686.4 | XP_002263722.1 | 1179 | *VvSTS46* | 16 |
| *VvSTS8* | XM_002263845.4 | XP_002263881.1 | 1179 | *VvSTS42* | 16 |
| *VvSTS9* | XM_010664202.4 | XP_010662504.1 | 1179 | *VvSTS25* | 16 |
| *VvSTS10* | XM_003634020.3 | XP_003634068.1 | 1179 | *VvSTS29* | 16 |
| *VvSTS11* | XM_002268806.4 | XP_002268842.2 | 1179 | *VvSTS27* | 16 |
| *VvSTS12* | XM_002263771.3 | XP_002263807.1 | 1179 | *VvSTS43* | 16 |
| *VvSTS13* | XM_003634017.3 | XP_003634065.1 | 1179 | *VvSTS37* | 16 |
| *VvSTS14* | NM_001281044.1 | NP_001267973.1 | 1179 | *VvSTS47* | 16 |
| *VvSTS15* | XM_002278349.3 | XP_002278385.1 | 1179 | *VvSTS9* | 16 |
| *VvSTS16* | XM_002278318.4 | XP_002278354.2 | 1179 | *VvSTS10* | 16 |
| *VvSTS17* | XM_002268720.4 | XP_002268756.1 | 1179 | *VvSTS21* | 16 |
| *VvSTS18* | XM_002269350.4 | XP_002269386.2 | 1179 | *VvSTS15* | 16 |
| *VvSTS19* | XM_002278447.4 | XP_002278483.1 | 1179 | *VvSTS7* | 16 |
| *VvSTS20* | XM_002264419.4 | XP_002264455.1 | 1179 | *VvSTS36* | 16 |
| *VvSTS21* | XM_003634016.3 | XP_003634064.1 | 1179 | *VvSTS38* | 16 |
| *VvSTS22* | XM_003634022.3 | XP_003634070.1 | 1179 | *VvSTS13* | 16 |
| *VvSTS23* | XM_003634023.3 | XP_003634071.1 | 1179 | *VvSTS23* | 16 |
| *VvSTS24* | XM_003634027.3 | XP_003634075.1 | 1179 | *VvSTS17* | 16 |
| *VvSTS25* | XM_003634024.3 | XP_003634072.1 | 1179 | *VvSTS16* | 16 |
| *VvSTS26* | XM_003634028.3 | XP_003634076.1 | 1179 | *VvSTS22* | 16 |
| *VvSTS27* | XM_003634019.3 | XP_003634067.1 | 1179 | *VvSTS30* | 16 |
| *VvSTS28* | XM_003634025.3 | XP_003634073.1 | 1179 | *VvSTS20* | 16 |
| *VvSTS29* | XM_003634021.3 | XP_003634069.1 | 1179 | *VvSTS28* | 16 |
| *VvSTS30* | XM_003634026.3 | XP_003634074.1 | 1179 | *VvSTS19* | 16 |
| *VvSTS31* | XM_002264953.4 | XP_002264989.1 | 1179 | *VvSTS12* | 16 |

*Vannozzi A, Dry IB, Fasoli M, Zenoni S, Lucchin M.  BMC Plant Biol. 2012;12:130

| **Gene** | **Forward primer (5'-3')** | **Reverse primer (5'-3')** |
| --- | --- | --- |
| *VvSTS1* | AATCACTCAAGGAAGAAAG | TTCACTCAATTTGTAGCC |
| *VvSTS2* | * (same as above) | * (same as above) |
| *VvSTS3* | AGAGAATGGTCCCTTTAACG | AACAATGACTCAATTACAATC |
| *VvSTS4* | * (same as above) | * (same as above) |
| *VvSTS5* | GCATTCCTACAGTTACAAATTAAG | CAATGACTCAAGTACAAATC |
| *VvSTS6* | * (same as above) | * (same as above) |
| *VvSTS7* | AGGTGAAGGATTGGATTGG | TTACATTAAGACATTGAAGGGT |
| *VvSTS8* | * (same as above) | * (same as above) |
| *VvSTS9* | GCATTCCTATGGTGACAAATTAA | TAGTTTCGGAGATAAATACCTTA |
| *VvSTS10* | * (same as above) | * (same as above) |
| *VvSTS11* | * (same as above) | * (same as above) |
| *VvSTS12* | ACAGGTGAAGGATTGGATTGG | CTTAATTTGAAACCGTAGGAATGC |
| *VvSTS13* | GGGTATTATTTGGTTTTGGG | AAAGACCATTCTCCCTTATT |
| *VvSTS14* | ATTCCTACGGTTACAAATTAAGTG | CAAAGAAAGTCTAACAATGACTTG |
| *VvSTS15* | GCATTCCTATGGTTTCTAATTGAG | ACACTATATCCACCAACAATCAC |
| *VvSTS16* | TTACAGAGGAGGTGCTAC | GCGATAACAGAATGACAA |
| *VvSTS17* | * (same as above) | * (same as above) |
| *VvSTS18* | * (same as above) | * (same as above) |
| *VvSTS19* | GATGAGATGAGAAGGAAATCAT | CGATGGTCAAGCCTGGTC |
| *VvSTS20* | GGACCAGGCTTAACCATCG | GACTCCAATTTGATACCGTAGAAC |
| *VvSTS21* | TGCCACGGGTACAAATTGAG | GAAGCCCTCCAGCAATCAGT |
| *VvSTS22* | CCAGGCTTGACCATTGAGACC | AGTCCTCCAAGAACGATAAATAAC |
| *VvSTS23* | * (same as above) | * (same as above) |
| *VvSTS24* | * (same as above) | * (same as above) |
| *VvSTS25* | TTATGGACGAGATGAGAAAG | AGGAATACTGTGGAGGAC |
| *VvSTS26* | * (same as above) | * (same as above) |
| *VvSTS27* | GGTGTCTTGTTTGGCTTTG | ACATGACTCATGAATAAGTTT |
| *VvSTS28* | * (same as above) | * (same as above) |
| *VvSTS29* | * (same as above) | * (same as above) |
| *VvSTS30* | * (same as above) | * (same as above) |
| *VvSTS31* | GGGTGTTTTGTTTGGCTTTG | ATGGTGGGAACTTGGACTCTC |

**Table S2.** List of primer pairs used in the present study for expression analysis of the *Vitis vinifera* stilbene synthase (*VvSTS*).

Asterisks denote identity among primers sequences (see the main text).

T**able S3**. *Vitis vinifera* gene sequences coding for chalcone synthase (*VvCHS*) identified in the NCBI database and used for the synthesis of oligonucleotides for expression analysis.

| **Gene** | **NCBI Reference Sequence (mRNA)** | **NCBI Reference Sequence (protein)** | **ORF (bp)** | **Chromosome location** | **Forward primer (5'-3')** | **Reverse primer (5'-3')** |
| --- | --- | --- | --- | --- | --- | --- |
| *VvCHS1* | XM_002263983.3 | XP_002264019.1 | 1170 | 5 | AGTCGGCTGAGGAAGGGC | CCAATACCAACAAGAGAAGG |
| *VvCHS2* | NM_001280950.1 | NP_001267879.1 | 1182 | 14 | GGAAAGGCAGCACAGGTG | CACCCAAGGATGACTACG |
| *VvCHS3* | NM_001281135.1 | NP_001268064.1 | 1365 | 14 | GGCGTTCTGTTTGGATTTG | CATTCCCATCTTCCCTTCAG |
| *VvCHS4* | XM_002276617.3 | XP_002276653.1 | 1179 | 3 | AGCTGAATGCTAGTAGGC | CCCACTCACCATCTCCCT |
| *VvCHS5* | XM_002276606.2 | XP_002276642.1 | 1170 | 15 | GCAATTCTGAACCGATTAG | ATTCTTCTCCTCCCTTCC |

**Table S4.** *Vitis vinifera* gene sequences coding for VvMYB14 and VvMYB15 transcription factors that specifically interact with the stilbene synthase promoter, identified in the NCBI database and used in the synthesis of oligonucleotides for expression analysis.

| **Gene** | **NCBI Reference Sequence (mRNA)** | **NCBI Reference Sequence (protein)** | **ORF (bp)** | **Chromosome location** | **Forward primer (5'-3')** | **Reverse primer (5'-3')** |
| --- | --- | --- | --- | --- | --- | --- |
| *VvMYB14* | NM_001281203.1 | NP_001268132.1 | 819 | 7 | GGAGAGCCTTGGGTATGG | GCAGGGTGTAGTAATGTCG |
| *VvMYB15* | XM_002285157.4 | XP_002285193.1 | 762 | 5 | GCACTGGCGTCAAGAATG | GTCCATAGGCGAGTTCCG |

**Table S5**. *Vitis vinifera* gene sequences coding for candidate reference genes identified in the NCBI database and used for the synthesis of oligonucleotides for the normalization of expression data in qRT-PCR analyses.

| **Gene** | **NCBI Reference Sequence** | **ORF (bp)** | **Chromosome location** | **Forward primer (5'-3')** | **Reverse primer (5'-3')** |
| --- | --- | --- | --- | --- | --- |
| *Vv60SPR* | XM_002270599 | 564 | 5 | GCTCCTCTCGGTCAGAACAC | GCTCTCTCAAACTTCCTTCC |
| *VvActin7* | XM_002282480 | 1134 | 4 | GTGCTTAGTGGTGGGTCAAC | TGCTGGAAGGTGCTGAGG |
| *VvVATP16* | XM_010649277 | 549 | 3 | CTAATGCGCAGCAGCCTA | TCGGGATGACAAGATAATGC |
| *VvUQCC* | XM_002264785 | 858 | 16 | TAGATGGATGAAGGACTTGG | AAGGATAGGCAACTACATTC |
| *VvSAND* | XM_002285134 | 1848 | 6 | TCAGTATGTATCTTCGGAGT | CCTTTACCCATTGACAGACC |
| *VvGAPDH* | XM_002263109 | 1014 | 17 | CCGTGTTCCTACTGTTGATG | CCTCTGACTCCTCCTTGATG |
| *VvEF1-α* | XM_002284888 | 1344 | 6 | GCAGGGTTTGTTAAGATGAT | TCCACGCTCTTGATGACTCC |
