# Supplementary material for: Transcriptional regulation of stilbene synthases in grapevine germplasm differentially susceptible to downy mildew
Source: BMC Plant Biol. 2019 Sep 14;19:404. doi: 10.1186/s12870-019-2014-5 (PMC6744718; doi:10.1186/s12870-019-2014-5)
Supplement: Supplementary file 2 — Table S6. Statistical evaluation of the differences (one-way ANOVA followed by Tukey test) among the relative expression levels of stilbene synthase (VvSTS) genes in the leaves of nine different grapevine genotypes (Gen, for genotypes acronyms, see the main text) after 0, 16, 24, 48, and 72 h from inoculation (hours post-inoculation, HPI) with Plasmopara viticola. For each of the stilbene synthase gene, the upper panel shows the statistical differences of the mean ± SD among the five sampling times for each grapevine genotype; the lower panel shows the statistical differences of the mean ± SD among the nine grapevine genotypes at each sampling time. Different letters denote statistically significant differences at p < 0.01; n.s., not statistically significant at the chosen probability threshold. (DOCX 59 kb) [file 12870_2019_2014_MOESM2_ESM.docx]

Manuscript title:

Transcriptional regulation of stilbene synthases in grapevine germplasm differentially susceptible to downy mildew.

Authors: Mario Ciaffi, Anna Rita Paolacci, Marco Paolocci, Enrica Alicandri, Valentina Bigini, Maurizio Badiani and Massimo Muganu.

The following Supplementary Table is available for the aforementioned manuscript in the present additional file 2:

**Table S6.**

Statistical evaluation of the differences (one-way ANOVA followed by Tukey test) among the relative expression levels of stilbene synthase (*VvSTS*) genes in the leaves of nine different *V. vinifera* genotypes (Gen, for genotypes acronyms, see the main text) after 0, 16, 24, 48, and 72 hours from inoculation (hours post-inoculation, HPI) with *Plasmopara viticola*. For each of the stilbene synthase genes, the upper panel shows the statistical differences of the mean ± SD among the five sampling times for each grapevine genotype; the lower panel shows the statistical differences of the mean ± SD among the nine grapevine genotypes at each sampling time. Different letters denote statistically significant differences at *p*< 0.01; n.s., not statistically significant at the chosen probability threshold.

***VvSTS1/2***

| **Gen** | **0 HPI** | **16 HPI** | **24 HPI** | **48 HPI** | **72 HPI** |
| --- | --- | --- | --- | --- | --- |
| CHA | 1.82 ± 0.33 abc | 2.19 ± 0.73 ab | 1.54 ± 0.27 a | 1.19 ± 0.42 a | 1.66 ± 0.41 a |
| ALE | 1.00 ± 0.16 a | 2.49 ± 0.49 ab | 2.81 ± 0.44 ab | 2.31 ± 0.41 ab | 1.79 ± 0.30 a |
| CAN | 1.20 ± 0.17 ab | 1.54 ± 0.28 a | 2.59 ± 0.59 a | 2.01 ± 0.25 a | 1.45 ± 0.30 a |
| TRE | 2.00 ± 0.33 bc | 1.64 ± 0.20 ab | 2.22 ± 0.41 a | 2.22 ± 0.36 a | 2.13 ± 0.39 ab |
| ROS | 2.36 ± 0.35 c | 2.15 ± 0.39 ab | 4.46 ± 0.64 bc | 3.83 ± 0.79 c | 3.48 ± 0.76 bc |
| ROM | 2.19 ± 0.32 c | 3.15 ± 0.37 b | 5.17 ± 0.92 c | 3.67 ± 0.65 bc | 3.44 ± 0.81 bc |
| SYL | 3.58 ± 0.43 d | 5.29 ± 1.78 c | 5.83 ± 1.36 c | 4.44 ± 0.75 c | 4.11 ± 0.86 c |
| ISA | 5.21 ± 0.73 e | 8.87 ± 1.59 d | 9.56 ± 2.24 d | 8.08 ± 1.25 d | 8.36 ± 1.98 d |
| SOL | 4.96 ± 0.65 e | 12.39 ± 2.24 e | 14.00 ± 2.39 e | 12.18 ± 2.54 e | 10.98 ± 2.11 e |

| **HPI** | **CHA** | **ALE** | **CAN** | **TRE** | **ROS** | **ROM** | **SYL** | **ISA** | **SOL** |
| --- | --- | --- | --- | --- | --- | --- | --- | --- | --- |
| 0 | 1.82 ± 0.33 ab | 1.00 ± 0.16 a | 1.20 ± 0.17 a | 2.00 ± 0.33 n.s. | 2.36 ± 0.35 a | 2.19 ± 0.32 a | 3.58 ± 0.43 a | 5.21 ± 0.73 a | 4.96 ± 0.65 a |
| 16 | 2.19 ± 0.73 b | 2.49 ± 0.49 c | 1.54 ± 0.28 ab | 1.64 ± 0.20 n.s. | 2.15 ± 0.39 a | 3.15 ± 0.37 b | 5.29 ± 1.78 bc | 8.87 ± 1.59 b | 12.39 ± 2.24 bc |
| 24 | 1.54 ± 0.27 a | 2.81 ± 0.44 c | 2.59 ± 0.59 c | 2.22 ± 0.41 n.s. | 4.46 ± 0.64 c | 5.17 ± 0.92 c | 5.83 ± 1.36 c | 9.56 ± 2.24 b | 14.00 ± 2.39 c |
| 48 | 1.19 ± 0.42 a | 2.31 ± 0.41 bc | 2.01 ± 0.25 bc | 2.22 ± 0.36 n.s. | 3.83 ± 0.79 bc | 3.67 ± 0.65 b | 4.44 ± 0.75 ab | 8.08 ± 1.25 b | 12.18 ± 2.54 bc |
| 72 | 1.66 ± 0.41 ab | 1.79 ± 0.30 b | 1.45 ± 0.3 ab | 2.13 ± 0.39 n.s. | 3.48 ± 0.76 b | 3.44 ± 0.81 b | 4.11 ± 0.86 ab | 8.36 ± 1.98 b | 10.98 ± 2.11 b |

***VvSTS3/4***

| **Gen** | **0 HPI** | **16 HPI** | **24 HPI** | **48 HPI** | **72 HPI** |
| --- | --- | --- | --- | --- | --- |
| CHA | 1.40 ± 0.14 a | 1.81 ± 0.46 a | 9.22 ± 1.37 a | 2.16 ± 0.33 a | 1.17 ± 0.17 a |
| ALE | 1.27 ± 0.28 a | 1.65 ± 0.23 a | 10.93 ± 1.65 a | 2.34 ± 0.42 a | 1.35 ± 0.23 a |
| CAN | 1.00 ± 0.21 a | 1.26 ± 0.27 a | 9.96 ± 1.49 a | 1.09 ± 0.16 a | 1.39 ± 0.29 a |
| TRE | 1.37 ± 0.23 a | 1.29 ± 0.25 a | 9.79 ± 1.62 a | 1.91 ± 0.30 a | 1.65 ± 0.39 a |
| ROS | 1.50 ± 0.30 a | 3.76 ± 0.57 b | 13.41 ± 2.78 ab | 6.27 ± 0.97 b | 10.68 ± 1.46 c |
| ROM | 1.67 ± 0.33 a | 3.06 ± 0.5 b | 17.70 ± 2.51 bc | 6.81 ± 1.00 b | 11.62 ± 1.57 c |
| SYL | 1.61 ± 0.41 a | 3.48 ± 0.63 b | 17.66 ± 2.27 bc | 2.99 ± 0.53 a | 5.13 ± 0.71 b |
| ISA | 2.94 ± 0.49 b | 5.21 ± 0.59 c | 21.37 ± 3.53 c | 9.55 ± 1.91 c | 15.06 ± 2.16 d |
| SOL | 2.81 ± 0.74 b | 4.70 ± 0.69 c | 33.33 ± 3.98 d | 16.53 ± 2.14 d | 23.58 ± 2.74 e |

| **HPI** | **CHA** | **ALE** | **CAN** | **TRE** | **ROS** | **ROM** | **SYL** | **ISA** | **SOL** |
| --- | --- | --- | --- | --- | --- | --- | --- | --- | --- |
| 0 | 1.40 ± 0.14 a | 1.27 ± 0.28 a | 1.00 ± 0.21 a | 1.37 ± 0.23 a | 1.50 ± 0.30 a | 1.67 ± 0.33 a | 1.61 ± 0.41 a | 2.94 ± 0.49 a | 2.81 ± 0.74 a |
| 16 | 1.81 ± 0.46 a | 1.65 ± 0.23 a | 1.26 ± 0.27 a | 1.29 ± 0.25 a | 3.76 ± 0.57 a | 3.06 ± 0.50 a | 3.48 ± 0.63 ab | 5.21 ± 0.59 a | 4.70 ± 0.69 a |
| 24 | 9.22 ± 1.37 b | 10.93 ± 1.65 b | 9.96 ± 1.49 b | 9.79 ± 1.62 b | 13.41 ± 2.78 d | 17.70 ± 2.51 d | 17.66 ± 2.27 c | 21.37 ± 3.53 d | 33.33 ± 3.98 d |
| 48 | 2.16 ± 0.33 a | 2.34 ± 0.42 a | 1.09 ± 0.16 a | 1.91 ± 0.30 a | 6.27 ± 0.97 b | 6.81 ± 1.00 b | 2.99 ± 0.53 a | 9.55 ± 1.91 b | 16.53 ± 2.14 b |
| 72 | 1.17 ± 0.17 a | 1.35 ± 0.23 a | 1.39 ± 0.29 a | 1.65 ± 0.39 a | 10.68 ± 1.46 c | 11.62 ± 1.57 c | 5.13 ± 0.71 b | 15.06 ± 2.16 c | 23.58 ± 2.74 c |

***VvSTS5/6***

| **Gen** | **0 HPI** | **16 HPI** | **24 HPI** | **48 HPI** | **72 HPI** |
| --- | --- | --- | --- | --- | --- |
| CHA | 1.98 ± 0.45 d | 1.73 ± 0.37 a | 9.21 ± 1.47 a | 2.43 ± 0.49 a | 2.59 ± 0.42 ab |
| ALE | 1.00 ± 0.22 a | 1.03 ± 0.22 a | 7.28 ± 1.02 a | 1.70 ± 0.30 a | 1.28 ± 0.25 a |
| CAN | 1.09 ± 0.18 ab | 1.67 ± 0.26 a | 7.16 ± 1.06 a | 1.16 ± 0.25 a | 1.99 ± 0.37 a |
| TRE | 1.67 ± 0.30 bcd | 1.63 ± 0.27 a | 8.51 ± 1.13 a | 1.76 ± 0.27 a | 2.01 ± 0.36 a |
| ROS | 1.85 ± 0.44 cde | 3.31 ± 0.56 bc | 14.60 ± 2.21 b | 5.50 ± 0.81 b | 7.76 ± 1.09 c |
| ROM | 1.28 ± 0.24 abc | 4.76 ± 0.81 de | 17.48 ± 2.07 b | 6.86 ± 0.96 b | 9.48 ± 1.53 c |
| SYL | 1.80 ± 0.32 cde | 2.81 ± 0.60 b | 14.73 ± 2.52 b | 1.38 ± 0.25 a | 4.64 ± 0.88 b |
| ISA | 2.32 ± 0.38 ef | 4.22 ± 0.62 cd | 22.34 ± 3.01 c | 9.07 ± 1.07 c | 15.51 ± 1.78 d |
| SOL | 2.79 ± 0.45 f | 5.27 ± 0.84 e | 31.48 ± 3.93 d | 15.70 ± 2.39 d | 21.92 ± 3.34 e |

| **HPI** | **CHA** | **ALE** | **CAN** | **TRE** | **ROS** | **ROM** | **SYL** | **ISA** | **SOL** |
| --- | --- | --- | --- | --- | --- | --- | --- | --- | --- |
| 0 | 1.98 ± 0.45 a | 1.00 ± 0.22 a | 1.09 ± 0.18 a | 1.67 ± 0.30 a | 1.85 ± 0.44 a | 1.28 ± 0.24 a | 1.80 ± 0.32 a | 2.32 ± 0.38 a | 2.79 ± 0.45 a |
| 16 | 1.73 ± 0.37 a | 1.03 ± 0.22 a | 1.67 ± 0.26 a | 1.63 ± 0.27 a | 3.31 ± 0.56 a | 4.76 ± 0.81 b | 2.81 ± 0.60 ab | 4.22 ± 0.62 a | 5.27 ± 0.84 a |
| 24 | 9.21 ± 1.47 b | 7.28 ± 1.02 b | 7.16 ± 1.06 b | 8.51 ± 1.13 b | 14.60 ± 2.21 d | 17.48 ± 2.07 d | 14.73 ± 2.52 c | 22.34 ± 3.01 d | 31.48 ± 3.93 d |
| 48 | 2.43 ± 0.49 a | 1.70 ± 0.30 a | 1.16 ± 0.25 a | 1.76 ± 0.27 a | 5.50 ± 0.81 b | 6.86 ± 0.96 b | 1.38 ± 0.25 a | 9.07 ± 1.07 b | 15.70 ± 2.39 b |
| 72 | 2.59 ± 0.42 a | 1.28 ± 0.25 a | 1.99 ± 0.37 a | 2.01 ± 0.36 a | 7.76 ± 1.09 c | 9.48 ± 1.53 c | 4.64 ± 0.88 b | 15.51 ± 1.78 c | 21.92 ± 3.34 c |

***VvSTS7/8***

| **Gen** | **0 HPI** | **16 HPI** | **24 HPI** | **48 HPI** | **72 HPI** |
| --- | --- | --- | --- | --- | --- |
| CHA | 1.30 ± 0.21 ab | 1.73 ± 0.51 abc | 1.25 ± 0.21 a | 1.55 ± 0.27 a | 1.84 ± 0.50 a |
| ALE | 1.00 ± 0.16 a | 1.13 ± 0.19 a | 2.30 ± 0.41 abc | 2.68 ± 0.58 a | 2.28 ± 0.52 a |
| CAN | 1.40 ± 0.25 abc | 1.84 ± 0.35 abc | 1.20 ± 0.34 a | 1.75 ± 0.40 a | 1.82 ± 0.56 a |
| TRE | 2.60 ± 0.37 ef | 2.06 ± 0.41 bcd | 2.55 ± 0.54 bc | 2.58 ± 0.54 a | 2.83 ± 0.65 a |
| ROS | 1.68 ± 0.27 bc | 1.51 ± 0.28 ab | 3.44 ± 0.74 c | 4.80 ± 0.80 b | 7.44 ± 1.04 b |
| ROM | 1.89 ± 0.27 cd | 2.77 ± 0.43 d | 3.35 ± 0.85 c | 6.43 ± 1.01 bc | 12.25 ± 1.66 c |
| SYL | 2.40 ± 0.38 def | 1.93 ± 0.39 bc | 2.11 ± 0.42 ab | 7.63 ± 1.28 c | 13.01 ± 1.83 c |
| ISA | 2.31 ± 0.42 de | 2.41 ± 0.4 cd | 2.09 ± 0.37 ab | 10.75 ± 1.55 d | 19.95 ± 2.99 d |
| SOL | 2.90 ± 0.46 f | 3.89 ± 0.65 e | 7.09 ± 1.24 d | 17.24 ± 2.17 e | 27.66 ± 3.44 e |

| **HPI** | **CHA** | **ALE** | **CAN** | **TRE** | **ROS** | **ROM** | **SYL** | **ISA** | **SOL** |
| --- | --- | --- | --- | --- | --- | --- | --- | --- | --- |
| 0 | 1.30 ± 0.21 n.s. | 1.00 ± 0.16 a | 1.40 ± 0.25 n.s. | 2.60 ± 0.37 n.s. | 1.68 ± 0.27 a | 1.89 ± 0.27 a | 2.40 ± 0.38 a | 2.31 ± 0.42 a | 2.90 ± 0.46 a |
| 16 | 1.73 ± 0.51 n.s. | 1.13 ± 0.19 a | 1.84 ± 0.35 n.s. | 2.06 ± 0.41 n.s. | 1.51 ± 0.28 a | 2.77 ± 0.43 a | 1.93 ± 0.39 a | 2.41 ± 0.40 a | 3.89 ± 0.65 a |
| 24 | 1.25 ± 0.21 n.s. | 2.30 ± 0.41 b | 1.20 ± 0.34 n.s. | 2.55 ± 0.54 n.s. | 3.44 ± 0.74 b | 3.35 ± 0.85 a | 2.11 ± 0.42 a | 2.09 ± 0.37 a | 7.09 ± 1.24 b |
| 48 | 1.55 ± 0.27 n.s. | 2.68 ± 0.58 b | 1.75 ± 0.40 n.s. | 2.58 ± 0.54 n.s. | 4.80 ± 0.80 c | 6.43 ± 1.01 b | 7.63 ± 1.28 b | 10.75 ± 1.55 b | 17.24 ± 2.17 c |
| 72 | 1.84 ± 0.50 n.s. | 2.28 ± 0.52 b | 1.82 ± 0.56 n.s. | 2.83 ± 0.65 n.s. | 7.44 ± 1.04 d | 12.25 ± 1.66 c | 13.01 ± 1.83 c | 19.95 ± 2.99 c | 27.66 ± 3.44 d |

***VvSTS9-11***

| **Gen** | **0 HPI** | **16 HPI** | **24 HPI** | **48 HPI** | **72 HPI** |
| --- | --- | --- | --- | --- | --- |
| CHA | 3.15 ± 0.53 bc | 3.70 ± 0.62 b | 3.99 ± 0.55 ab | 3.92 ± 0.66 a | 3.97 ± 0.64 ab |
| ALE | 1.00 ± 0.14 a | 2.57 ± 0.54 a | 4.21 ± 0.67 ab | 2.56 ± 0.42 a | 2.25 ± 0.42 a |
| CAN | 2.49 ± 0.45 b | 2.96 ± 0.46 ab | 2.98 ± 0.44 a | 3.34 ± 0.63 a | 3.29 ± 0.57 a |
| TRE | 2.85 ± 0.39 bc | 3.82 ± 0.45 b | 3.54 ± 0.40 a | 3.49 ± 0.56 a | 7.41 ± 1.15 bc |
| ROS | 2.37 ± 0.39 b | 2.43 ± 0.34 a | 3.63 ± 0.49 ab | 7.88 ± 1.07 b | 10.44 ± 1.46 cd |
| ROM | 2.56 ± 0.32 bc | 2.93 ± 0.34 ab | 6.87 ± 0.92 cd | 10.02 ± 1.51 b | 15.03 ± 2.16 e |
| SYL | 3.43 ± 0.64 cd | 3.93 ± 0.86 b | 5.04 ± 1.30 b | 7.73 ± 1.47 b | 13.65 ± 1.83 de |
| ISA | 4.17 ± 0.75 de | 5.16 ± 0.87 c | 6.61 ± 0.90 c | 13.77 ± 1.63 c | 24.68 ± 2.68 f |
| SOL | 4.41 ± 0.77 e | 5.21 ± 0.81 c | 8.14 ± 1.08 d | 21.39 ± 3.28 d | 30.73 ± 4.19 g |

| **HPI** | **CHA** | **ALE** | **CAN** | **TRE** | **ROS** | **ROM** | **SYL** | **ISA** | **SOL** |
| --- | --- | --- | --- | --- | --- | --- | --- | --- | --- |
| 0 | 3.15 ± 0.53 n.s. | 1.00 ± 0.14 a | 2.49 ± 0.45 n.s. | 2.85 ± 0.39 a | 2.37 ± 0.39 a | 2.56 ± 0.32 a | 3.43 ± 0.64 a | 4.17 ± 0.75 a | 4.41 ± 0.77 a |
| 16 | 3.70 ± 0.62 n.s. | 2.57 ± 0.54 b | 2.96 ± 0.46 n.s. | 3.82 ± 0.45 a | 2.43 ± 0.34 a | 2.93 ± 0.34 a | 3.93 ± 0.86 a | 5.16 ± 0.87 a | 5.21 ± 0.81 a |
| 24 | 3.99 ± 0.55 n.s. | 4.21 ± 0.67 c | 2.98 ± 0.44 n.s. | 3.54 ± 0.40 a | 3.63 ± 0.49 a | 6.87 ± 0.92 b | 5.04 ± 1.30 a | 6.61 ± 0.90 a | 8.14 ± 1.08 a |
| 48 | 3.92 ± 0.66 n.s. | 2.56 ± 0.42 b | 3.34 ± 0.63 n.s. | 3.49 ± 0.56 a | 7.88 ± 1.07 b | 10.02 ± 1.51 c | 7.73 ± 1.47 b | 13.77 ± 1.63 b | 21.39 ± 3.28 b |
| 72 | 3.97 ± 0.64 n.s. | 2.25 ± 0.42 b | 3.29 ± 0.57 n.s. | 7.41 ± 1.15 b | 10.44 ± 1.46 c | 15.03 ± 2.16 d | 13.65 ± 1.83 c | 24.68 ± 2.68 c | 30.73 ± 4.19 c |

***VvSTS12***

| **Gen** | **0 HPI** | **16 HPI** | **24 HPI** | **48 HPI** | **72 HPI** |
| --- | --- | --- | --- | --- | --- |
| CHA | 2.63 ± 0.41 cd | 3.52 ± 0.42 b | 8.41 ± 1.29 a | 4.93 ± 0.88 bc | 3.52 ± 0.46 a |
| ALE | 1.00 ± 0.16 a | 1.57 ± 0.29 a | 7.66 ± 1.19 a | 2.06 ± 0.32 a | 1.83 ± 0.30 a |
| CAN | 1.70 ± 0.23 ab | 2.31 ± 0.42 ab | 6.78 ± 1.08 a | 2.41 ± 0.34 a | 2.42 ± 0.31 a |
| TRE | 2.42 ± 0.45 bc | 2.12 ± 0.36 a | 4.97 ± 0.80 a | 2.15 ± 0.28 a | 2.44 ± 0.37 a |
| ROS | 2.36 ± 0.44 bc | 5.02 ± 0.69 c | 14.62 ± 1.98 b | 4.07 ± 0.45 b | 8.49 ± 1.40 b |
| ROM | 1.88 ± 0.23 bc | 6.55 ± 1.19 d | 18.36 ± 2.43 c | 6.32 ± 0.89 c | 10.39 ± 1.39 b |
| SYL | 4.17 ± 0.62 e | 7.66 ± 1.01 de | 15.69 ± 1.93 bc | 5.49 ± 1.04 bc | 8.11 ± 1.65 b |
| ISA | 3.31 ± 0.38 d | 7.26 ± 0.85 de | 25.59 ± 2.34 d | 8.51 ± 1.20 d | 17.28 ± 1.76 c |
| SOL | 4.49 ± 0.70 e | 7.96 ± 0.82 e | 31.19 ± 3.37 e | 9.85 ± 1.45 d | 21.30 ± 2.47 d |

| **HPI** | **CHA** | **ALE** | **CAN** | **TRE** | **ROS** | **ROM** | **SYL** | **ISA** | **SOL** |
| --- | --- | --- | --- | --- | --- | --- | --- | --- | --- |
| 0 | 2.63 ± 0.41 a | 1.00 ± 0.16 a | 1.70 ± 0.23 a | 2.42 ± 0.45 a | 2.36 ± 0.44 a | 1.88 ± 0.23 a | 4.17 ± 0.62 a | 3.31 ± 0.38 a | 4.49 ± 0.70 a |
| 16 | 3.52 ± 0.42 a | 1.57 ± 0.29 ab | 2.31 ± 0.42 a | 2.12 ± 0.36 a | 5.02 ± 0.69 b | 6.55 ± 1.19 b | 7.66 ± 1.01 bc | 7.26 ± 0.85 b | 7.96 ± 0.82 b |
| 24 | 8.41 ± 1.29 c | 7.66 ± 1.19 c | 6.78 ± 1.08 b | 4.97 ± 0.80 b | 14.62 ± 1.98 d | 18.36 ± 2.43 d | 15.69 ± 1.93 d | 25.59 ± 2.34 d | 31.19 ± 3.37 d |
| 48 | 4.93 ± 0.88 b | 2.06 ± 0.32 b | 2.41 ± 0.34 a | 2.15 ± 0.28 a | 4.07 ± 0.45 ab | 6.32 ± 0.89 b | 5.49 ± 1.04 ab | 8.51 ± 1.20 b | 9.85 ± 1.45 b |
| 72 | 3.52 ± 0.46 a | 1.83 ± 0.3 ab | 2.42 ± 0.31 a | 2.44 ± 0.37 a | 8.49 ± 1.40 c | 10.39 ± 1.39 c | 8.11 ± 1.65 c | 17.28 ± 1.76 c | 21.30 ± 2.47 c |

***VvSTS13***

| **Gen** | **0 HPI** | **16 HPI** | **24 HPI** | **48 HPI** | **72 HPI** |
| --- | --- | --- | --- | --- | --- |
| CHA | 1.81 ± 0.34 bc | 2.47 ± 0.56 ab | 5.53 ± 1.54 a | 1.15 ± 0.28 a | 1.03 ± 0.21 a |
| ALE | 1.00 ± 0.16 a | 2.54 ± 0.41 bc | 5.43 ± 0.81 a | 1.47 ± 0.21 a | 2.40 ± 0.37 a |
| CAN | 1.52 ± 0.26 ab | 1.90 ± 0.41 ab | 5.28 ± 1.16 a | 1.00 ± 0.24 a | 1.32 ± 0.38 a |
| TRE | 1.41 ± 0.23 ab | 1.31 ± 0.29 a | 6.29 ± 1.18 a | 1.22 ± 0.22 a | 2.56 ± 0.44 a |
| ROS | 1.78 ± 0.28 bc | 2.79 ± 0.48 bc | 12.06 ± 1.43 b | 5.74 ± 0.97 b | 7.41 ± 1.52 b |
| ROM | 1.72 ± 0.19 bc | 3.69 ± 0.60 c | 16.68 ± 2.18 c | 7.77 ± 1.13 c | 11.57 ± 1.60 c |
| SYL | 2.14 ± 0.38 c | 2.33 ± 0.83 ab | 14.12 ± 2.61 bc | 1.14 ± 0.22 a | 6.81 ± 0.99 b |
| ISA | 2.80 ± 0.47 d | 6.82 ± 0.99 d | 25.69 ± 3.36 d | 11.61 ± 1.68 d | 16.91 ± 1.89 d |
| SOL | 3.19 ± 0.57 d | 7.57 ± 1.05 d | 34.11 ± 4.75 e | 20.38 ± 2.45 e | 28.34 ± 3.16 e |

| **HPI** | **CHA** | **ALE** | **CAN** | **TRE** | **ROS** | **ROM** | **SYL** | **ISA** | **SOL** |
| --- | --- | --- | --- | --- | --- | --- | --- | --- | --- |
| 0 | 1.81 ± 0.34 ab | 1.00 ± 0.16 a | 1.52 ± 0.26 a | 1.41 ± 0.23 a | 1.78 ± 0.28 a | 1.72 ± 0.19 a | 2.14 ± 0.38 a | 2.80 ± 0.47 a | 3.19 ± 0.57 a |
| 16 | 2.47 ± 0.56 b | 2.54 ± 0.41 b | 1.90 ± 0.41 a | 1.31 ± 0.29 a | 2.79 ± 0.48 a | 3.69 ± 0.60 a | 2.33 ± 0.83 a | 6.82 ± 0.99 b | 7.57 ± 1.05 a |
| 24 | 5.53 ± 1.54 c | 5.43 ± 0.81 c | 5.28 ± 1.16 b | 6.29 ± 1.18 c | 12.06 ± 1.43 c | 16.68 ± 2.18 d | 14.12 ± 2.61 c | 25.69 ± 3.36 e | 34.11 ± 4.75 d |
| 48 | 1.15 ± 0.28 a | 1.47 ± 0.21 a | 1.00 ± 0.24 a | 1.22 ± 0.22 a | 5.74 ± 0.97 b | 7.77 ± 1.13 b | 1.14 ± 0.22 a | 11.61 ± 1.68 c | 20.38 ± 2.45 b |
| 72 | 1.03 ± 0.21 a | 2.40 ± 0.37 b | 1.32 ± 0.38 a | 2.56 ± 0.44 b | 7.41 ± 1.52 b | 11.57 ± 1.60 c | 6.81 ± 0.99 b | 16.91 ± 1.89 d | 28.34 ± 3.16 c |

***VvSTS14***

| **Gen** | **0 HPI** | **16 HPI** | **24 HPI** | **48 HPI** | **72 HPI** |
| --- | --- | --- | --- | --- | --- |
| CHA | 1.79 ± 0.32 bc | 2.87 ± 0.55 cd | 7.06 ± 1.27 a | 1.34 ± 0.28 a | 2.31 ± 0.47 ab |
| ALE | 1.21 ± 0.23 ab | 2.37 ± 0.49 bcd | 5.94 ± 0.86 a | 2.10 ± 0.33 a | 2.21 ± 0.39 ab |
| CAN | 1.00 ± 0.22 a | 1.21 ± 0.19 a | 5.14 ± 0.69 a | 1.51 ± 0.19 a | 1.63 ± 0.23 a |
| TRE | 1.71 ± 0.31 bc | 1.83 ± 0.32 ab | 5.82 ± 0.92 a | 2.24 ± 0.43 a | 2.12 ± 0.53 ab |
| ROS | 1.42 ± 0.28 ab | 1.99 ± 0.30 abc | 13.82 ± 1.82 b | 4.52 ± 0.56 b | 6.87 ± 1.01 c |
| ROM | 1.32 ± 0.22 ab | 1.86 ± 0.33 ab | 19.11 ± 2.44 c | 7.16 ± 0.70 c | 10.06 ± 1.10 d |
| SYL | 2.27 ± 0.38 cd | 3.00 ± 0.88 d | 15.58 ± 2.68 bc | 1.78 ± 0.37 a | 4.26 ± 0.68 b |
| ISA | 2.98 ± 0.46 e | 4.26 ± 0.75 e | 31.36 ± 4.84 d | 11.90 ± 1.72 d | 16.68 ± 2.65 e |
| SOL | 2.76 ± 0.57 de | 7.59 ± 0.71 f | 27.16 ± 3.62 d | 16.65 ± 2.12 e | 19.28 ± 2.63 f |

| **HPI** | **CHA** | **ALE** | **CAN** | **TRE** | **ROS** | **ROM** | **SYL** | **ISA** | **SOL** |
| --- | --- | --- | --- | --- | --- | --- | --- | --- | --- |
| 0 | 1.79 ± 0.32 ab | 1.21 ± 0.23 a | 1.00 ± 0.22 a | 1.71 ± 0.31 a | 1.42 ± 0.28 a | 1.32 ± 0.22 a | 2.27 ± 0.38 ab | 2.98 ± 0.46 a | 2.76 ± 0.57 a |
| 16 | 2.87 ± 0.55 b | 2.37 ± 0.49 b | 1.21 ± 0.19 ab | 1.83 ± 0.32 a | 1.99 ± 0.30 a | 1.86 ± 0.33 a | 3.00 ± 0.88 ab | 4.26 ± 0.75 a | 7.59 ± 0.71 b |
| 24 | 7.06 ± 1.27 c | 5.94 ± 0.86 c | 5.14 ± 0.69 c | 5.82 ± 0.92 b | 13.82 ± 1.82 d | 19.11 ± 2.44 d | 15.58 ± 2.68 c | 31.36 ± 4.84 d | 27.16 ± 3.62 d |
| 48 | 1.34 ± 0.28 a | 2.10 ± 0.33 b | 1.51 ± 0.19 ab | 2.24 ± 0.43 a | 4.52 ± 0.56 b | 7.16 ± 0.70 b | 1.78 ± 0.37 a | 11.90 ± 1.72 b | 16.65 ± 2.12 c |
| 72 | 2.31 ± 0.47 ab | 2.21 ± 0.39 b | 1.63 ± 0.23 b | 2.12 ± 0.53 a | 6.87 ± 1.01 c | 10.06 ± 1.10 c | 4.26 ± 0.68 b | 16.68 ± 2.65 c | 19.28 ± 2.63 c |

***VvSTS15***

| **Gen** | **0 HPI** | **16 HPI** | **24 HPI** | **48 HPI** | **72 HPI** |
| --- | --- | --- | --- | --- | --- |
| CHA | 1.58 ± 0.24 bc | 1.86 ± 0.25 c | 2.43 ± 0.32 ab | 2.32 ± 0.36 a | 3.11 ± 0.52 a |
| ALE | 1.00 ± 0.17 a | 2.66 ± 0.55 e | 2.61 ± 0.61 ab | 2.53 ± 0.39 a | 2.27 ± 0.34 a |
| CAN | 1.65 ± 0.26 bc | 2.53 ± 0.38 de | 2.91 ± 0.40 abc | 2.69 ± 0.36 a | 3.08 ± 0.53 a |
| TRE | 1.81 ± 0.33 c | 1.21 ± 0.24 ab | 1.73 ± 0.46 a | 1.90 ± 0.41 a | 3.60 ± 0.74 a |
| ROS | 1.35 ± 0.27 abc | 1.73 ± 0.26 bc | 2.85 ± 0.44 abc | 5.78 ± 0.92 bc | 7.29 ± 1.29 b |
| ROM | 1.18 ± 0.17 ab | 1.06 ± 0.18 a | 2.91 ± 0.57 abc | 5.21 ± 0.69 b | 9.04 ± 1.44 b |
| SYL | 1.60 ± 0.24 bc | 2.49 ± 0.28 de | 3.96 ± 0.66 c | 7.31 ± 0.94 c | 13.72 ± 2.02 c |
| ISA | 2.55 ± 0.42 d | 2.17 ± 0.35 cde | 3.22 ± 0.63 bc | 10.59 ± 1.15 d | 21.98 ± 1.91 d |
| SOL | 2.75 ± 0.51 d | 1.96 ± 0.43 cd | 6.75 ± 1.43 d | 14.67 ± 2.36 e | 27.16 ± 3.54 e |

| **HPI** | **CHA** | **ALE** | **CAN** | **TRE** | **ROS** | **ROM** | **SYL** | **ISA** | **SOL** |
| --- | --- | --- | --- | --- | --- | --- | --- | --- | --- |
| 0 | 1.58 ± 0.24 a | 1.00 ± 0.17 a | 1.65 ± 0.26 a | 1.81 ± 0.33 a | 1.35 ± 0.27 a | 1.18 ± 0.17 a | 1.60 ± 0.24 a | 2.55 ± 0.42 a | 2.75 ± 0.51 a |
| 16 | 1.86 ± 0.25 ab | 2.66 ± 0.55 b | 2.53 ± 0.38 b | 1.21 ± 0.24 a | 1.73 ± 0.26 ab | 1.06 ± 0.18 a | 2.49 ± 0.28 ab | 2.17 ± 0.35 a | 1.96 ± 0.43 a |
| 24 | 2.43 ± 0.32 b | 2.61 ± 0.61 b | 2.91 ± 0.40 b | 1.73 ± 0.46 a | 2.85 ± 0.44 b | 2.91 ± 0.57 b | 3.96 ± 0.66 b | 3.22 ± 0.63 a | 6.75 ± 1.43 b |
| 48 | 2.32 ± 0.36 b | 2.53 ± 0.39 b | 2.69 ± 0.36 b | 1.90 ± 0.41 a | 5.78 ± 0.92 c | 5.21 ± 0.69 c | 7.31 ± 0.94 c | 10.59 ± 1.15 b | 14.67 ± 2.36 c |
| 72 | 3.11 ± 0.52 c | 2.27 ± 0.34 b | 3.08 ± 0.53 b | 3.60 ± 0.74 b | 7.29 ± 1.29 d | 9.04 ± 1.44 d | 13.72 ± 2.02 d | 21.98 ± 1.91 c | 27.16 ± 3.54 d |

***VvSTS16-18***

| **Gen** | **0 HPI** | **16 HPI** | **24 HPI** | **48 HPI** | **72 HPI** |
| --- | --- | --- | --- | --- | --- |
| CHA | 2.15 ± 0.41 b | 1.67 ± 0.30 bc | 2.30 ± 0.43 bc | 2.04 ± 0.36 a | 4.44 ± 1.24 a |
| ALE | 1.31 ± 0.17 a | 1.63 ± 0.44 abc | 2.74 ± 0.40 c | 3.31 ± 0.50 b | 4.32 ± 0.73 a |
| CAN | 1.16 ± 0.17 a | 1.00 ± 0.15 a | 1.53 ± 0.24 ab | 1.71 ± 0.32 a | 3.85 ± 0.74 a |
| TRE | 2.54 ± 0.33 bc | 2.42 ± 0.35 d | 2.20 ± 0.34 bc | 2.58 ± 0.33 ab | 4.24 ± 0.81 a |
| ROS | 1.38 ± 0.18 a | 1.34 ± 0.26 abc | 1.19 ± 0.17 a | 5.55 ± 0.62 c | 8.71 ± 0.92 b |
| ROM | 1.36 ± 0.20 a | 1.19 ± 0.19 ab | 1.35 ± 0.20 a | 7.10 ± 0.48 d | 11.48 ± 1.09 bc |
| SYL | 2.61 ± 0.47 bc | 1.98 ± 0.37 cd | 2.87 ± 0.59 c | 6.53 ± 0.42 cd | 12.29 ± 1.78 c |
| ISA | 2.63 ± 0.34 bc | 2.47 ± 0.37 d | 4.10 ± 0.57 d | 9.06 ± 0.96 e | 22.12 ± 2.73 d |
| SOL | 2.93 ± 0.37 c | 3.97 ± 0.61 e | 5.11 ± 0.68 e | 12.95 ± 1.36 f | 29.28 ± 2.58 e |

| **HPI** | **CHA** | **ALE** | **CAN** | **TRE** | **ROS** | **ROM** | **SYL** | **ISA** | **SOL** |
| --- | --- | --- | --- | --- | --- | --- | --- | --- | --- |
| 0 | 2.15 ± 0.41 a | 1.31 ± 0.17 a | 1.16 ± 0.17 ab | 2.54 ± 0.33 a | 1.38 ± 0.18 a | 1.36 ± 0.20 a | 2.61 ± 0.47 a | 2.63 ± 0.34 a | 2.93 ± 0.37 a |
| 16 | 1.67 ± 0.30 a | 1.63 ± 0.44 a | 1.00 ± 0.15 a | 2.42 ± 0.35 a | 1.34 ± 0.26 a | 1.19 ± 0.19 a | 1.98 ± 0.37 a | 2.47 ± 0.37 a | 3.97 ± 0.61 a |
| 24 | 2.30 ± 0.43 a | 2.74 ± 0.40 b | 1.53 ± 0.24 ab | 2.20 ± 0.34 a | 1.19 ± 0.17 a | 1.35 ± 0.20 a | 2.87 ± 0.59 a | 4.10 ± 0.57 a | 5.11 ± 0.68 a |
| 48 | 2.04 ± 0.36 a | 3.31 ± 0.50 b | 1.71 ± 0.32 b | 2.58 ± 0.33 a | 5.55 ± 0.62 b | 7.10 ± 0.48 b | 6.53 ± 0.42 b | 9.06 ± 0.96 b | 12.95 ± 1.36 b |
| 72 | 4.44 ± 1.24 b | 4.32 ± 0.73 c | 3.85 ± 0.74 c | 4.24 ± 0.81 b | 8.71 ± 0.92 c | 11.48 ± 1.09 c | 12.29 ± 1.78 c | 22.12 ± 2.73 c | 29.28 ± 2.58 c |

***VvSTS19***

| **Gen** | **0 HPI** | **16 HPI** | **24 HPI** | **48 HPI** | **72 HPI** |
| --- | --- | --- | --- | --- | --- |
| CHA | 2.12 ± 0.35 c | 2.24 ± 0.51 c | 2.69 ± 0.53 c | 3.04 ± 0.61 ab | 3.62 ± 0.86 a |
| ALE | 1.11 ± 0.14 a | 2.14 ± 0.45 c | 2.01 ± 0.38 bc | 2.52 ± 0.36 a | 3.08 ± 0.57 a |
| CAN | 1.38 ± 0.19 ab | 1.09 ± 0.16 a | 1.00 ± 0.16 a | 2.30 ± 0.35 a | 2.62 ± 0.41 a |
| TRE | 1.93 ± 0.28 c | 1.97 ± 0.53 bc | 2.64 ± 0.62 c | 2.36 ± 0.52 a | 3.39 ± 0.74 a |
| ROS | 1.88 ± 0.26 bc | 1.14 ± 0.23 a | 1.63 ± 0.34 ab | 4.50 ± 0.71 bc | 6.78 ± 1.14 b |
| ROM | 1.28 ± 0.17 a | 1.27 ± 0.18 ab | 2.36 ± 0.42 bc | 5.59 ± 0.54 cd | 10.07 ± 1.16 c |
| SYL | 1.97 ± 0.26 c | 1.76 ± 0.38 abc | 1.42 ± 0.35 ab | 6.77 ± 0.68 d | 10.17 ± 1.25 c |
| ISA | 2.14 ± 0.30 c | 1.88 ± 0.29 bc | 4.38 ± 0.51 d | 11.27 ± 1.39 e | 18.21 ± 2.17 d |
| SOL | 3.12 ± 0.49 d | 4.01 ± 0.61 d | 7.42 ± 1.02 e | 14.35 ± 1.92 f | 25.89 ± 2.91 e |

| **HPI** | **CHA** | **ALE** | **CAN** | **TRE** | **ROS** | **ROM** | **SYL** | **ISA** | **SOL** |
| --- | --- | --- | --- | --- | --- | --- | --- | --- | --- |
| 0 | 2.12 ± 0.35 a | 1.11 ± 0.14 a | 1.38 ± 0.19 a | 1.93 ± 0.28 a | 1.88 ± 0.26 a | 1.28 ± 0.17 a | 1.97 ± 0.26 a | 2.14 ± 0.30 a | 3.12 ± 0.49 a |
| 16 | 2.24 ± 0.51 a | 2.14 ± 0.45 b | 1.09 ± 0.16 a | 1.97 ± 0.53 a | 1.14 ± 0.23 a | 1.27 ± 0.18 a | 1.76 ± 0.38 a | 1.88 ± 0.29 a | 4.01 ± 0.61 a |
| 24 | 2.69 ± 0.53 ab | 2.01 ± 0.38 b | 1.00 ± 0.16 a | 2.64 ± 0.62 ab | 1.63 ± 0.34 a | 2.36 ± 0.42 b | 1.42 ± 0.35 a | 4.38 ± 0.51 b | 7.42 ± 1.02 b |
| 48 | 3.04 ± 0.61 ab | 2.52 ± 0.36 bc | 2.30 ± 0.35 b | 2.36 ± 0.52 a | 4.50 ± 0.71 b | 5.59 ± 0.54 c | 6.77 ± 0.68 b | 11.27 ± 1.39 c | 14.35 ± 1.92 c |
| 72 | 3.62 ± 0.86 b | 3.08 ± 0.57 c | 2.62 ± 0.41 b | 3.39 ± 0.74 b | 6.78 ± 1.14 c | 10.07 ± 1.16 d | 10.17 ± 1.25 c | 18.21 ± 2.17 d | 25.89 ± 2.91 d |

***VvSTS20***

| **Gen** | **0 HPI** | **16 HPI** | **24 HPI** | **48 HPI** | **72 HPI** |
| --- | --- | --- | --- | --- | --- |
| CHA | 1.71 ± 0.37 b | 1.51 ± 0.34 ab | 1.77 ± 0.39 ab | 1.61 ± 0.34 n.s. | 1.81 ± 0.39 n.s. |
| ALE | 1.00 ± 0.23 a | 1.37 ± 0.33 ab | 1.39 ± 0.34 ab | 1.48 ± 0.39 n.s. | 1.35 ± 0.35 n.s. |
| CAN | 1.38 ± 0.20 ab | 1.33 ± 0.23 ab | 1.26 ± 0.17 a | 1.35 ± 0.24 n.s. | 1.46 ± 0.25 n.s. |
| TRE | 1.25 ± 0.25 ab | 1.23 ± 0.21 a | 1.41 ± 0.28 ab | 1.27 ± 0.16 n.s. | 1.35 ± 0.17 n.s. |
| ROS | 1.35 ± 0.27 ab | 1.31 ± 0.20 ab | 1.50 ± 0.17 ab | 1.64 ± 0.28 n.s. | 1.52 ± 0.34 n.s. |
| ROM | 1.42 ± 0.30 ab | 1.60 ± 0.24 ab | 1.83 ± 0.35 b | 1.61 ± 0.36 n.s. | 1.75 ± 0.38 n.s. |
| SYL | 1.72 ± 0.35 b | 1.41 ± 0.42 ab | 1.38 ± 0.34 ab | 1.29 ± 0.33 n.s. | 1.36 ± 0.27 n.s. |
| ISA | 1.63 ± 0.39 b | 1.73 ± 0.30 ab | 1.73 ± 0.39 ab | 1.48 ± 0.31 n.s. | 1.67 ± 0.25 n.s. |
| SOL | 1.75 ± 0.32 b | 1.78 ± 0.37 b | 1.92 ± 0.25 b | 1.74 ± 0.41 n.s. | 1.72 ± 0.26 n.s. |

| **HPI** | **CHA** | **ALE** | **CAN** | **TRE** | **ROS** | **ROM** | **SYL** | **ISA** | **SOL** |
| --- | --- | --- | --- | --- | --- | --- | --- | --- | --- |
| 0 | 1.71 ± 0.37 n.s. | 1.00 ± 0.23 n.s. | 1.38 ± 0.20 n.s. | 1.25 ± 0.25 n.s. | 1.35 ± 0.27 n.s. | 1.42 ± 0.30 n.s. | 1.72 ± 0.35 n.s. | 1.63 ± 0.39 n.s. | 1.75 ± 0.32 n.s. |
| 16 | 1.51 ± 0.34 n.s. | 1.37 ± 0.33 n.s. | 1.33 ± 0.23 n.s. | 1.23 ± 0.21 n.s. | 1.31 ± 0.20 n.s. | 1.60 ± 0.24 n.s. | 1.41 ± 0.42 n.s. | 1.73 ± 0.30 n.s. | 1.78 ± 0.37 n.s. |
| 24 | 1.77 ± 0.39 n.s. | 1.39 ± 0.34 n.s. | 1.26 ± 0.17 n.s. | 1.41 ± 0.28 n.s. | 1.50 ± 0.17 n.s. | 1.83 ± 0.35 n.s. | 1.38 ± 0.34 n.s. | 1.73 ± 0.39 n.s. | 1.92 ± 0.25 n.s. |
| 48 | 1.61 ± 0.34 n.s. | 1.48 ± 0.39 n.s. | 1.35 ± 0.24 n.s. | 1.27 ± 0.16 n.s. | 1.64 ± 0.28 n.s. | 1.61 ± 0.36 n.s. | 1.29 ± 0.33 n.s. | 1.48 ± 0.31 n.s. | 1.74 ± 0.41 n.s. |
| 72 | 1.81 ± 0.39 n.s. | 1.35 ± 0.35 n.s. | 1.46 ± 0.25 n.s. | 1.35 ± 0.17 n.s. | 1.52 ± 0.34 n.s. | 1.75 ± 0.38 n.s. | 1.36 ± 0.27 n.s. | 1.67 ± 0.25 n.s. | 1.72 ± 0.26 n.s. |

***VvSTS21***

| **Gen** | **0 HPI** | **16 HPI** | **24 HPI** | **48 HPI** | **72 HPI** |
| --- | --- | --- | --- | --- | --- |
| CHA | 1.51 ± 0.3 bcd | 1.44 ± 0.37 abc | 1.01 ± 0.26 a | 1.40 ± 0.39 a | 1.63 ± 0.48 ab |
| ALE | 1.00 ± 0.15 a | 1.37 ± 0.19 ab | 1.38 ± 0.21 ab | 1.64 ± 0.23 ab | 1.35 ± 0.25 a |
| CAN | 1.12 ± 0.17 ab | 1.45 ± 0.15 abc | 1.69 ± 0.28 bc | 1.69 ± 0.31 ab | 1.95 ± 0.31 ab |
| TRE | 1.52 ± 0.23 bcd | 1.29 ± 0.21 a | 1.49 ± 0.23 bc | 1.68 ± 0.29 ab | 1.76 ± 0.38 ab |
| ROS | 1.20 ± 0.18 ab | 1.78 ± 0.26 bcd | 1.53 ± 0.19 bc | 1.96 ± 0.31 ab | 1.92 ± 0.28 ab |
| ROM | 1.29 ± 0.21 abc | 1.70 ± 0.19 abcd | 1.47 ± 0.21 ab | 1.68 ± 0.29 ab | 2.01 ± 0.32 ab |
| SYL | 1.76 ± 0.29 d | 1.87 ± 0.27 cd | 1.48 ± 0.26 b | 1.89 ± 0.42 ab | 2.04 ± 0.52 b |
| ISA | 1.72 ± 0.38 cd | 2.06 ± 0.28 d | 1.94 ± 0.36 c | 2.09 ± 0.40 b | 2.02 ± 0.26 b |
| SOL | 1.95 ± 0.35 d | 1.95 ± 0.33 d | 1.68 ± 0.29 bc | 2.01 ± 0.28 b | 2.26 ± 0.44 b |

| **HPI** | **CHA** | **ALE** | **CAN** | **TRE** | **ROS** | **ROM** | **SYL** | **ISA** | **SOL** |
| --- | --- | --- | --- | --- | --- | --- | --- | --- | --- |
| 0 | 1.51 ± 0.30 ab | 1.00 ± 0.15 a | 1.12 ± 0.17 a | 1.52 ± 0.23 ab | 1.20 ± 0.18 a | 1.29 ± 0.21 a | 1.76 ± 0.29 n.s. | 1.72 ± 0.38 n.s. | 1.95 ± 0.35 ab |
| 16 | 1.44 ± 0.37 ab | 1.37 ± 0.19 b | 1.45 ± 0.15 ab | 1.29 ± 0.21 a | 1.78 ± 0.26 bc | 1.70 ± 0.19 ab | 1.87 ± 0.27 n.s. | 2.06 ± 0.28 n.s. | 1.95 ± 0.33 ab |
| 24 | 1.01 ± 0.26 a | 1.38 ± 0.21 b | 1.69 ± 0.28 ab | 1.49 ± 0.23 ab | 1.53 ± 0.19 ab | 1.47 ± 0.21 a | 1.48 ± 0.26 n.s. | 1.94 ± 0.36 n.s. | 1.68 ± 0.29 a |
| 48 | 1.40 ± 0.39 ab | 1.64 ± 0.23 b | 1.69 ± 0.31 bc | 1.68 ± 0.29 ab | 1.96 ± 0.31 c | 1.68 ± 0.29 ab | 1.89 ± 0.42 n.s. | 2.09 ± 0.40 n.s. | 2.01 ± 0.28 ab |
| 72 | 1.63 ± 0.48 b | 1.35 ± 0.25 ab | 1.95 ± 0.31 c | 1.76 ± 0.38 b | 1.92 ± 0.28 bc | 2.01 ± 0.32 b | 2.04 ± 0.52 n.s. | 2.02 ± 0.26 n.s. | 2.26 ± 0.44 b |

***VvSTS22-24***

| **Gen** | **0 HPI** | **16 HPI** | **24 HPI** | **48 HPI** | **72 HPI** |
| --- | --- | --- | --- | --- | --- |
| CHA | 1.89 ± 0.30 bc | 2.36 ± 0.67 a | 3.29 ± 0.88 a | 1.73 ± 0.51 a | 1.20 ± 0.32 a |
| ALE | 1.00 ± 0.17 a | 1.99 ± 0.35 a | 2.72 ± 0.46 a | 2.40 ± 0.41 ab | 1.91 ± 0.32 ab |
| CAN | 1.49 ± 0.35 ab | 2.24 ± 0.44 a | 3.56 ± 0.68 ab | 1.23 ± 0.27 a | 1.58 ± 0.37 ab |
| TRE | 1.91 ± 0.24 bcd | 2.68 ± 0.63 a | 3.03 ± 0.65 a | 1.64 ± 0.32 a | 1.40 ± 0.28 ab |
| ROS | 2.53 ± 0.38 cd | 5.81 ± 0.92 b | 8.08 ± 1.37 cd | 4.13 ± 0.78 bc | 3.77 ± 0.69 cd |
| ROM | 2.72 ± 0.53 d | 4.99 ± 0.79 b | 10.11 ± 1.73 d | 5.10 ± 0.63 c | 4.02 ± 0.76 d |
| SYL | 2.72 ± 0.61 d | 4.83 ± 1.01 b | 6.65 ± 1.47 bc | 1.93 ± 0.40 a | 2.60 ± 0.59 bc |
| ISA | 3.88 ± 0.45 e | 11.30 ± 1.95 c | 16.31 ± 2.41 e | 11.35 ± 1.68 d | 7.15 ± 1.10 e |
| SOL | 4.92 ± 0.78 f | 11.30 ± 1.78 c | 21.92 ± 3.79 f | 14.57 ± 2.31 e | 9.09 ± 1.56 f |

| **HPI** | **CHA** | **ALE** | **CAN** | **TRE** | **ROS** | **ROM** | **SYL** | **ISA** | **SOL** |
| --- | --- | --- | --- | --- | --- | --- | --- | --- | --- |
| 0 | 1.89 ± 0.30 ab | 1.00 ± 0.17 a | 1.49 ± 0.35 a | 1.91 ± 0.24 a | 2.53 ± 0.38 a | 2.72 ± 0.53 a | 2.72 ± 0.61 a | 3.88 ± 0.45 a | 4.92 ± 0.78 a |
| 16 | 2.36 ± 0.67 bc | 1.99 ± 0.35 b | 2.24 ± 0.44 b | 2.68 ± 0.63 b | 5.81 ± 0.92 c | 4.99 ± 0.79 b | 4.83 ± 1.01 b | 11.30 ± 1.95 c | 11.30 ± 1.78 b |
| 24 | 3.29 ± 0.88 c | 2.72 ± 0.46 c | 3.56 ± 0.68 c | 3.03 ± 0.65 b | 8.08 ± 1.37 d | 10.11 ± 1.73 c | 6.65 ± 1.47 c | 16.31 ± 2.41 d | 21.92 ± 3.79 c |
| 48 | 1.73 ± 0.51 ab | 2.40 ± 0.41 bc | 1.23 ± 0.27 a | 1.64 ± 0.32 a | 4.13 ± 0.78 b | 5.10 ± 0.63 b | 1.93 ± 0.40 a | 11.35 ± 1.68 c | 14.57 ± 2.31 a |
| 72 | 1.20 ± 0.32 a | 1.91 ± 0.32 b | 1.58 ± 0.37 ab | 1.40 ± 0.28 a | 3.77 ± 0.69 ab | 4.02 ± 0.76 ab | 2.60 ± 0.59 a | 7.15 ± 1.10 b | 9.09 ± 1.56 a |

***VvSTS25/26***

| **Gen** | **0 HPI** | **16 HPI** | **24 HPI** | **48 HPI** | **72 HPI** |
| --- | --- | --- | --- | --- | --- |
| CHA | 1.59 ± 0.25 abc | 1.84 ± 0.48 a | 3.03 ± 0.62 a | 1.21 ± 0.26 ab | 1.13 ± 0.23 a |
| ALE | 1.00 ± 0.15 a | 2.21 ± 0.36 a | 3.87 ± 0.83 a | 1.74 ± 0.32 ab | 1.84 ± 0.35 ab |
| CAN | 1.46 ± 0.30 ab | 1.71 ± 0.34 a | 2.83 ± 0.56 a | 1.13 ± 0.22 a | 1.76 ± 0.41 ab |
| TRE | 2.40 ± 0.42 cd | 2.44 ± 0.48 a | 3.71 ± 1.04 a | 1.84 ± 0.34 ab | 1.46 ± 0.32 a |
| ROS | 2.30 ± 0.38 bcd | 5.06 ± 0.79 bc | 8.89 ± 1.39 b | 3.42 ± 0.65 b | 3.56 ± 0.63 b |
| ROM | 2.98 ± 0.53 de | 6.48 ± 0.89 cd | 11.41 ± 1.95 b | 8.20 ± 1.34 c | 6.43 ± 1.21 c |
| SYL | 3.71 ± 0.63 ef | 4.37 ± 0.79 b | 10.10 ± 1.49 b | 2.35 ± 0.42 ab | 2.58 ± 0.43 ab |
| ISA | 4.38 ± 0.75 fg | 8.01 ± 1.39 d | 16.84 ± 2.26 c | 12.95 ± 2.10 d | 10.05 ± 1.85 d |
| SOL | 4.85 ± 0.73 g | 13.08 ± 2.04 e | 22.17 ± 2.73 d | 18.04 ± 2.63 e | 13.87 ± 1.96 e |

| **HPI** | **CHA** | **ALE** | **CAN** | **TRE** | **ROS** | **ROM** | **SYL** | **ISA** | **SOL** |
| --- | --- | --- | --- | --- | --- | --- | --- | --- | --- |
| 0 | 1.59 ± 0.25 ab | 1.00 ± 0.15 a | 1.46 ± 0.30 a | 2.40 ± 0.42 ab | 2.30 ± 0.38 a | 2.98 ± 0.53 a | 3.71 ± 0.63 ab | 4.38 ± 0.75 a | 4.85 ± 0.73 a |
| 16 | 1.84 ± 0.48 b | 2.21 ± 0.36 b | 1.71 ± 0.34 a | 2.44 ± 0.48 b | 5.06 ± 0.79 b | 6.48 ± 0.89 b | 4.37 ± 0.79 b | 8.01 ± 1.39 b | 13.08 ± 2.04 b |
| 24 | 3.03 ± 0.62 c | 3.87 ± 0.83 c | 2.83 ± 0.56 b | 3.71 ± 1.04 c | 8.89 ± 1.39 c | 11.41 ± 1.95 c | 10.10 ± 1.49 c | 16.84 ± 2.26 d | 22.17 ± 2.73 d |
| 48 | 1.21 ± 0.26 ab | 1.74 ± 0.32 ab | 1.13 ± 0.22 a | 1.84 ± 0.34 ab | 3.42 ± 0.65 a | 8.20 ± 1.34 b | 2.35 ± 0.42 a | 12.95 ± 2.10 c | 18.04 ± 2.63 c |
| 72 | 1.13 ± 0.23 a | 1.84 ± 0.35 b | 1.76 ± 0.41 a | 1.46 ± 0.32 a | 3.56 ± 0.63 a | 6.43 ± 1.21 b | 2.58 ± 0.43 a | 10.05 ± 1.85 b | 13.87 ± 1.96 b |

***VvSTS27-30***

| **Gen** | **0 HPI** | **16 HPI** | **24 HPI** | **48 HPI** | **72 HPI** |
| --- | --- | --- | --- | --- | --- |
| CHA | 2.05 ± 0.35 ab | 2.63 ± 0.45 a | 4.10 ± 0.51 a | 1.58 ± 0.19 ab | 2.19 ± 0.41 ab |
| ALE | 1.25 ± 0.17 a | 3.75 ± 0.61 ab | 4.75 ± 0.72 a | 2.62 ± 0.4 abc | 3.32 ± 0.54 bc |
| CAN | 1.59 ± 0.29 a | 2.33 ± 0.40 a | 3.37 ± 0.41 a | 1.00 ± 0.19 a | 1.19 ± 0.16 a |
| TRE | 2.20 ± 0.57 ab | 2.12 ± 0.54 a | 3.84 ± 0.99 a | 1.64 ± 0.38 ab | 1.68 ± 0.38 ab |
| ROS | 2.89 ± 0.46 b | 5.04 ± 0.76 bc | 11.49 ± 1.74 b | 4.97 ± 0.76 c | 5.54 ± 0.84 d |
| ROM | 4.12 ± 0.39 c | 8.67 ± 0.75 d | 15.96 ± 1.97 c | 10.08 ± 0.84 d | 7.50 ± 0.97 e |
| SYL | 5.29 ± 0.78 d | 6.96 ± 1.09 cd | 13.32 ± 1.61 bc | 3.95 ± 0.64 bc | 4.06 ± 0.69 cd |
| ISA | 5.29 ± 0.98 d | 14.52 ± 2.49 e | 24.80 ± 3.33 d | 14.06 ± 2.8 e | 10.50 ± 2.01 f |
| SOL | 6.34 ± 0.95 d | 16.61 ± 2.28 e | 31.05 ± 3.16 e | 19.49 ± 2.61 f | 11.96 ± 1.71 f |

| **HPI** | **CHA** | **ALE** | **CAN** | **TRE** | **ROS** | **ROM** | **SYL** | **ISA** | **SOL** |
| --- | --- | --- | --- | --- | --- | --- | --- | --- | --- |
| 0 | 2.05 ± 0.35 ab | 1.25 ± 0.17 a | 1.59 ± 0.29 b | 2.20 ± 0.57 a | 2.89 ± 0.46 a | 4.12 ± 0.39 a | 5.29 ± 0.78 ab | 5.29 ± 0.98 a | 6.34 ± 0.95 a |
| 16 | 2.63 ± 0.45 b | 3.75 ± 0.61 c | 2.33 ± 0.40 c | 2.12 ± 0.54 a | 5.04 v 0.76 b | 8.67 ± 0.75 bc | 6.96 ± 1.09 b | 14.52 ± 2.49 b | 16.61 ± 2.28 c |
| 24 | 4.10 ± 0.51 c | 4.75 ± 0.72 d | 3.37 ± 0.41 d | 3.84 ± 0.99 b | 11.49 ± 1.74 c | 15.96 ± 1.97 d | 13.32 ± 1.61 c | 24.80 ± 3.33 c | 31.05 ± 3.16 d |
| 48 | 1.58 ± 0.19 a | 2.62 ± 0.40 b | 1.00 ± 0.19 a | 1.64 ± 0.38 a | 4.97 ± 0.76 b | 10.08 ± 0.84 c | 3.95 ± 0.64 a | 14.06 ± 2.80 b | 19.49 ± 2.61 c |
| 72 | 2.19 ± 0.41 ab | 3.32 ± 0.54 bc | 1.19 ± 0.16 ab | 1.68 ± 0.38 a | 5.54 ± 0.84 b | 7.50 ± 0.97 b | 4.06 ± 0.69 a | 10.50 ± 2.01 b | 11.96 ± 1.71 b |

***VvSTS31***

| **Gen** | **0 HPI** | **16 HPI** | **24 HPI** | **48 HPI** | **72 HPI** |
| --- | --- | --- | --- | --- | --- |
| CHA | 1.71 ± 0.27 a | 2.29 ± 0.43 a | 3.18 ± 0.50 a | 1.97 ± 0.38 a | 1.17 ± 0.17 a |
| ALE | 1.38 ± 0.21 a | 3.45 ± 0.48 ab | 4.04 ± 0.72 a | 2.35 ± 0.33 ab | 1.75 ± 0.34 a |
| CAN | 1.00 ± 0.16 a | 1.75 ± 0.31 a | 3.81 ± 0.58 a | 1.93 ± 0.35 a | 1.29 ± 0.19 a |
| TRE | 1.92 ± 0.37 ab | 1.83 ± 0.37 a | 3.59 ± 0.59 a | 2.32 ± 0.28 ab | 1.31 ± 0.19 a |
| ROS | 2.72 ± 0.43 bc | 5.19 ± 0.74 bc | 10.76 ± 1.52 b | 6.85 ± 0.87 cd | 5.11 ± 0.80 b |
| ROM | 3.30 ± 0.53 c | 5.84 ± 0.93 c | 11.95 ± 2.06 b | 9.19 ± 1.43 d | 8.44 ± 1.34 c |
| SYL | 4.29 ± 0.68 d | 5.84 ± 1.26 c | 13.96 ± 3.01 b | 5.19 ± 0.74 bc | 2.96 ± 0.62 a |
| ISA | 6.73 ± 0.71 e | 11.72 ± 1.82 d | 25.53 ± 3.59 c | 17.59 ± 2.83 e | 12.18 ± 2.06 d |
| SOL | 7.09 ± 0.98 e | 20.83 ± 2.86 e | 34.00 ± 4.70 d | 20.89 ± 3.81 f | 13.96 ± 1.57 d |

| **HPI** | **CHA** | **ALE** | **CAN** | **TRE** | **ROS** | **ROM** | **SYL** | **ISA** | **SOL** |
| --- | --- | --- | --- | --- | --- | --- | --- | --- | --- |
| 0 | 1.71 ± 0.27 ab | 1.38 ± 0.21 a | 1.00 ± 0.16 a | 1.92 ± 0.37 ab | 2.72 ± 0.43 a | 3.30 ± 0.53 a | 4.29 ± 0.68 ab | 6.73 ± 0.71 a | 7.09 ± 0.98 a |
| 16 | 2.29 ± 0.43 b | 3.45 ± 0.48 c | 1.75 ± 0.31 bc | 1.83 ± 0.37 ab | 5.19 ± 0.74 b | 5.84 ± 0.93 b | 5.84 ± 1.26 b | 11.72 ± 1.82 b | 20.83 ± 2.86 c |
| 24 | 3.18 ± 0.50 c | 4.04 ± 0.72 c | 3.81 ± 0.58 d | 3.59 ± 0.59 c | 10.76 ± 1.52 d | 11.95 ± 2.06 d | 13.96 ± 3.01 c | 25.53 ± 3.59 d | 34.00 ± 4.70 d |
| 48 | 1.97 ± 0.38 b | 2.35 ± 0.33 b | 1.93 ± 0.35 c | 2.32 ± 0.28 b | 6.85 ± 0.87 c | 9.19 ± 1.43 c | 5.19 ± 0.74 ab | 17.59 ± 2.83 c | 20.89 ± 3.81 c |
| 72 | 1.17 ± 0.17 a | 1.75 ± 0.34 ab | 1.29 ± 0.19 ab | 1.31 ± 0.19 a | 5.11 ± 0.80 b | 8.44 ± 1.34 c | 2.96 ± 0.62 a | 12.18 ± 2.06 b | 13.96 ± 1.57 b |
