# Supplementary material for: Transcriptional regulation of stilbene synthases in grapevine germplasm differentially susceptible to downy mildew
Source: BMC Plant Biol. 2019 Sep 14;19:404. doi: 10.1186/s12870-019-2014-5 (PMC6744718; doi:10.1186/s12870-019-2014-5)
Supplement: Supplementary file 3 — Table S7. Statistical evaluation of the differences (one-way ANOVA followed by Tukey test) among the relative expression levels of MYB transcription factors genes (VvMYB14 and VvMYB15) in the leaves of nine different Vitis vinifera genotypes (Gen, for genotypes acronyms, see the text) after 0, 16, 24, 48, and 72 h from inoculation (hours post-inoculation, HPI) with Plasmopara viticola. For each of the MYB transcription factors genes, the upper panel shows the statistical differences of the mean ± SD among the five sampling times for each grapevine genotype; the lower panel shows the statistical differences of the mean ± SD among the nine grapevine genotypes at each sampling time. Different letters denote statistically significant differences at p < 0.01; n.s., not statistically significant at the chosen probability threshold. Table S8. Statistical evaluation of the differences (one-way ANOVA followed by Tukey test) among the relative expression levels of chalcone synthase genes (VvCHS1–3) in the leaves of nine different grapevine genotypes (Gen, for genotypes acronyms, see the text) after 0, 16, 24, 48, and 72 h from inoculation (hours post-inoculation, HPI) with Plasmopara viticola. For each of the chalcone synthase gene, the upper panel shows the statistical differences of the mean ± SD among the five sampling times for each grapevine genotype; the lower panel shows the statistical differences of the mean ± SD among the nine grapevine genotypes at each sampling time. Different letters denote statistically significant differences at p < 0.01; n.s., not statistically significant at the chosen probability threshold. (DOCX 28 kb) [file 12870_2019_2014_MOESM3_ESM.docx]

Manuscript title:

Transcriptional regulation of stilbene synthases in grapevine germplasm differentially susceptible to downy mildew.

Authors: Mario Ciaffi, Anna Rita Paolacci, Marco Paolocci, Enrica Alicandri, Valentina Bigini, Maurizio Badiani and Massimo Muganu.

The following Supplementary Tables are available for the aforementioned manuscript in the present additional file 3:

**Table S7.**

Statistical evaluation of the differences (one-way ANOVA followed by Tukey test) among the relative expression levels of MYB transcription factors genes (*VvMYB14* and *VvMYB15*) in the leaves of nine different *Vitis vinifera* genotypes (Gen, for genotypes acronyms, see the text) after 0, 16, 24, 48, and 72 hours from inoculation (hours post-inoculation, HPI) with *Plasmopara viticola*. For each of the MYB transcription factors genes, the upper panel shows the statistical differences of the mean ± SD among the five sampling times for each grapevine genotype; the lower panel shows the statistical differences of the mean ± SD among the nine grapevine genotypes at each sampling time. Different letters denote statistically significant differences at *p*< 0.01; n.s., not statistically significant at the chosen probability threshold.

**Table S8.**

Statistical evaluation of the differences (one-way ANOVA followed by Tukey test) among the relative expression levels of chalcone synthase genes (*VvCHS1-3*) in the leaves of nine different grapevine genotypes (Gen, for genotypes acronyms, see the text) after 0, 16, 24, 48, and 72 hours from inoculation (hours post-inoculation, HPI) with *Plasmopara viticola*. For each of the chalcone synthase gene, the upper panel shows the statistical differences of the mean ± SD among the five sampling times for each grapevine genotype; the lower panel shows the statistical differences of the mean ± SD among the nine grapevine genotypes at each sampling time. Different letters denote statistically significant differences at *p*< 0.01; n.s., not statistically significant at the chosen probability threshold.

**Table S7.**

Statistical evaluation of the differences (one-way ANOVA followed by Tukey test) among the relative expression levels of MYB transcription factors genes (*VvMYB14* and *VvMYB15*) in the leaves of nine different *Vitis vinifera* genotypes (Gen, for genotypes acronyms, see the text) after 0, 16, 24, 48, and 72 hours from inoculation (hours post-inoculation, HPI) with *Plasmopara viticola*. For each of the MYB transcription factors genes, the upper panel shows the statistical differences of the mean ± SD among the five sampling times for each grapevine genotype; the lower panel shows the statistical differences of the mean ± SD among the nine grapevine genotypes at each sampling time. Different letters denote statistically significant differences at *p*< 0.01; n.s., not statistically significant at the chosen probability threshold.

***VvMYB14***

| **Gen** | **0 HPI** | **16 HPI** | **24 HPI** | **48 HPI** | **72 HPI** |
| --- | --- | --- | --- | --- | --- |
| CHA | 2.73 ± 0.45 ab | 2.58 ± 0.40 a | 7.57 ± 1.17 d | 9.47 ± 1.40 c | 10.80 ± 1.20 d |
| ALE | 1.80 ± 0.32 a | 5.25 ± 0.75 c | 6.76 ± 0.93 cd | 8.00 ± 1.10 c | 8.76 ± 1.31 c |
| CAN | 2.76 ± 0.45 ab | 4.79 ± 0.67 bc | 7.95 ± 0.96 d | 11.29 ± 1.22 d | 12.06 ± 1.65 d |
| TRE | 5.25 ± 0.83 c | 10.13 ± 0.91 e | 9.69 ± 1.38 e | 13.88 ± 1.55 e | 14.31 ± 1.64 e |
| ROS | 3.16 ± 0.53 b | 5.10 ± 0.75 c | 5.64 ± 0.64 bc | 2.56 ± 0.42 a | 2.93 ± 0.52 ab |
| ROM | 2.02 ± 0.36 a | 3.91 ± 0.64 b | 4.42 ± 0.75 b | 2.28 ± 0.41 a | 2.28 ± 0.41 ab |
| SYL | 4.63 ± 0.72 c | 6.91 ± 0.72 d | 7.75 ± 0.82 d | 5.04 ± 0.63 b | 4.03 ± 0.52 b |
| ISA | 3.10 ± 0.46 b | 2.30 ± 0.39 a | 1.76 ± 0.29 a | 1.00 ± 0.17 a | 1.14 ± 0.21 a |
| SOL | 4.35 ± 0.64 c | 2.03 ± 0.37 a | 1.24 ± 0.21 a | 1.03 ± 0.18 a | 1.23 ± 0.16 a |

| **HPI** | **CHA** | **ALE** | **CAN** | **TRE** | **ROS** | **ROM** | **SYL** | **ISA** | **SOL** |
| --- | --- | --- | --- | --- | --- | --- | --- | --- | --- |
| 0 | 1.27 ± 0.19 a | 1.00 ± 0.21 a | 1.37 ± 0.24 a | 1.39 ± 0.25 a | 1.29 ± 0.25 a | 1.17 ± 0.20 a | 1.40 ± 0.28 a | 1.20 ± 0.19 a | 1.57 ± 0.42 a |
| 16 | 3.02 ± 0.47 c | 2.82 ± 0.29 c | 3.18 ± 0.41 c | 2.76 ± 0.42 c | 5.41 ± 0.64 d | 7.21 ± 0.77 c | 8.33 ± 1.05 d | 15.07 ± 1.59 d | 20.98 ± 2.40 d |
| 24 | 2.05 ± 0.33 b | 1.72 ± 0.27 b | 2.13 ± 0.30 b | 1.92 ± 0.31 ab | 3.20 ± 0.48 b | 3.69 ± 0.53 b | 3.00 ± 0.52 b | 5.69 ± 0.65 b | 8.99 ± 1.06 b |
| 48 | 2.17 ± 0.37 b | 1.55 ± 0.24 b | 1.99 ± 0.33 b | 1.84 ± 0.27 ab | 4.33 ± 0.58 c | 6.30 ± 0.82 c | 4.96 ± 0.62 c | 8.89 ± 1.05 c | 12.98 ± 1.87 c |
| 72 | 2.47 ± 0.41 bc | 1.72 ± 0.28 b | 1.90 ± 0.32 ab | 2.17 ± 0.38 b | 4.50 ± 0.73 cd | 6.64 ± 0.74 c | 5.50 ± 0.61 c | 9.58 ± 1.17 c | 14.09 ± 2.01 c |

**Table S7 - continued**

***VvMYB15***

| **Gen** | **0 HPI** | **16 HPI** | **24 HPI** | **48 HPI** | **72 HPI** |
| --- | --- | --- | --- | --- | --- |
| CHA | 1.29 ± 0.29 ab | 1.02 ± 0.23 a | 1.16 ± 0.23 a | 3.00 ± 0.62 a | 3.81 ± 0.64 a |
| ALE | 1.00 ± 0.19 a | 1.24 ± 0.26 a | 1.88 ± 0.43 a | 2.41 ± 0.37 a | 2.88 ± 0.64 a |
| CAN | 1.53 ± 0.29 b | 1.57 ± 0.25 a | 2.32 ± 0.33 a | 3.09 ± 0.55 a | 3.31 ± 0.71 a |
| TRE | 1.47 ± 0.32 ab | 1.11 ± 0.18 a | 1.64 ± 0.35 a | 2.94 ± 0.40 a | 3.19 ± 0.57 a |
| ROS | 1.27 ± 0.26 ab | 1.72 ± 0.28 a | 3.75 ± 0.68 b | 5.71 ± 1.08 b | 6.56 ± 1.23 b |
| ROM | 1.19 ± 0.17 ab | 1.77 ± 0.39 a | 4.61 ± 0.64 bc | 5.99 ± 0.82 b | 6.73 ± 1.02 b |
| SYL | 1.16 ± 0.20 ab | 1.53 ± 0.23 a | 5.15 ± 0.68 c | 6.52 ± 1.00 b | 7.45 ± 1.13 b |
| ISA | 1.34 ± 0.28 ab | 3.33 ± 0.65 b | 7.70 ± 1.01 d | 10.21 ± 1.13 c | 12.08 ± 1.61 c |
| SOL | 1.57 ± 0.35 b | 4.13 ± 0.86 c | 10.73 ± 1.53 e | 14.09 ± 1.46 d | 15.50 ± 1.87 d |

| **HPI** | **CHA** | **ALE** | **CAN** | **TRE** | **ROS** | **ROM** | **SYL** | **ISA** | **SOL** |
| --- | --- | --- | --- | --- | --- | --- | --- | --- | --- |
| 0 | 1.29 ± 0.29 a | 1.00 ± 0.19 a | 1.53 ± 0.29 a | 1.47 ± 0.32 a | 1.27 ± 0.26 a | 1.19 ± 0.17 a | 1.16 ± 0.20 a | 1.34 ± 0.28 a | 1.57 ± 0.35 a |
| 16 | 1.02 ± 0.23 a | 1.24 ± 0.26 ab | 1.57 ± 0.25 ab | 1.11 ± 0.18 a | 1.72 ± 0.28 a | 1.77 ± 0.39 a | 1.53 ± 0.23 a | 3.33 ± 0.65 b | 4.13 ± 0.86 b |
| 24 | 1.16 ± 0.23 a | 1.88 ± 0.43 bc | 2.32 ± 0.33 bc | 1.64 ± 0.35 a | 3.75 ± 0.68 b | 4.61 ± 0.64 b | 5.15 ± 0.68 b | 7.70 ± 1.01 c | 10.73 ± 1.53 c |
| 48 | 3.00 ± 0.62 b | 2.41 ± 0.37 cd | 3.09 ± 0.55 cd | 2.94 ± 0.40 b | 5.71 ± 1.08 c | 5.99 ± 0.82 c | 6.52 ± 1.00 c | 10.21 ± 1.13 d | 14.09 ± 1.46 d |
| 72 | 3.81 ± 0.64 c | 2.88 ± 0.64 d | 3.31 ± 0.71 d | 3.19 ± 0.57 b | 6.56 ± 1.23 c | 6.73 ± 1.02 c | 7.45 ± 1.13 c | 12.08 ± 1.61 e | 15.50 ± 1.87 d |

**Table S8.**

Statistical evaluation of the differences (one-way ANOVA followed by Tukey test) among the relative expression levels of chalcone synthase genes (*VvCHS1-3*) in the leaves of nine different grapevine genotypes (Gen, for genotypes acronyms, see the text) after 0, 16, 24, 48, and 72 hours from inoculation (hours post-inoculation, HPI) with *Plasmopara viticola*. For each of the chalcone synthase gene, the upper panel shows the statistical differences of the mean ± SD among the five sampling times for each grapevine genotype; the lower panel shows the statistical differences of the mean ± SD among the nine grapevine genotypes at each sampling time. Different letters denote statistically significant differences at *p*< 0.01; n.s., not statistically significant at the chosen probability threshold.

***VvCHS1***

| **Gen** | **0 HPI** | **16 HPI** | **24 HPI** | **48 HPI** | **72 HPI** |
| --- | --- | --- | --- | --- | --- |
| CHA | 2.73 ± 0.45 ab | 2.58 ± 0.40 a | 7.57 ± 1.17 d | 9.47 ± 1.40 c | 10.80 ± 1.20 d |
| ALE | 1.80 ± 0.32 a | 5.25 ± 0.75 c | 6.76 ± 0.93 cd | 8.00 ± 1.10 c | 8.76 ± 1.31 c |
| CAN | 2.76 ± 0.45 ab | 4.79 ± 0.67 bc | 7.95 ± 0.96 d | 11.29 ± 1.22 d | 12.06 ± 1.65 d |
| TRE | 5.25 ± 0.83 c | 10.13 ± 0.91 e | 9.69 ± 1.38 e | 13.88 ± 1.55 e | 14.31 ± 1.64 e |
| ROS | 3.16 ± 0.53 b | 5.10 ± 0.75 c | 5.64 ± 0.64 bc | 2.56 ± 0.42 a | 2.93 ± 0.52 ab |
| ROM | 2.02 ± 0.36 a | 3.91 ± 0.64 b | 4.42 ± 0.75 b | 2.28 ± 0.41 a | 2.28 ± 0.41 ab |
| SYL | 4.63 ± 0.72 c | 6.91 ± 0.72 d | 7.75 ± 0.82 d | 5.04 ± 0.63 b | 4.03 ± 0.52 b |
| ISA | 3.10 ± 0.46 b | 2.30 ± 0.39 a | 1.76 ± 0.29 a | 1.00 ± 0.17 a | 1.14 ± 0.21 a |
| SOL | 4.35 ± 0.64 c | 2.03 ± 0.37 a | 1.24 ± 0.21 a | 1.03 ± 0.18 a | 1.23 ± 0.16 a |

| **HPI** | **CHA** | **ALE** | **CAN** | **TRE** | **ROS** | **ROM** | **SYL** | **ISA** | **SOL** |
| --- | --- | --- | --- | --- | --- | --- | --- | --- | --- |
| 0 | 2.73 ± 0.45 a | 1.80 ± 0.32 a | 2.76 ± 0.45 a | 5.25 ± 0.83 a | 3.16 ± 0.53 a | 2.02 ± 0.36 a | 4.63 ± 0.72 a | 3.10 ± 0.46 d | 4.35 ± 0.64 c |
| 16 | 2.58 ± 0.40 a | 5.25 ± 0.75 b | 4.79 ± 0.67 b | 10.13 ± 0.91 b | 5.10 ± 0.75 b | 3.91 ± 0.64 b | 6.91 ± 0.72 b | 2.30 ± 0.39 c | 2.03 ± 0.37 b |
| 24 | 7.57 ± 1.17 b | 6.76 ± 0.93 bc | 7.95 ± 0.96 c | 9.69 ± 1.38 b | 5.64 ± 0.64 b | 4.42 ± 0.75 b | 7.75 ± 0.82 b | 1.76 ± 0.29 b | 1.24 ± 0.21 a |
| 48 | 9.47 ± 1.40 c | 8.00 ± 1.10 cd | 11.29 ± 1.22 d | 13.88 ± 1.55 c | 2.56 ± 0.42 a | 2.28 ± 0.41 a | 5.04 ± 0.63 a | 1.00 ± 0.17 a | 1.03 ± 0.18 a |
| 72 | 10.80 ± 1.20 c | 8.76 ± 1.31 d | 12.06 ± 1.65 d | 14.31 ± 1.64 c | 2.93 ± 0.52 a | 2.28 ± 0.41 a | 4.03 ± 0.52 a | 1.14 ± 0.21 a | 1.23 ± 0.16 a |

**Table S8 - continued**

***VvCHS2***

| **Gen** | **0 HPI** | **16 HPI** | **24 HPI** | **48 HPI** | **72 HPI** |
| --- | --- | --- | --- | --- | --- |
| CHA | 3.41 ± 0.56 d | 3.49 ± 0.55 cde | 6.30 ± 0.86 f | 5.77 ± 0.83 e | 6.66 ± 0.98 d |
| ALE | 1.74 ± 0.27 ab | 3.46 ± 0.58 cde | 5.14 ± 0.63 e | 4.22 ± 0.70 d | 5.92 ± 0.87 cd |
| CAN | 2.06 ± 0.40 abc | 3.00 ± 0.47 bcd | 4.62 ± 0.73 de | 4.67 ± 0.71 d | 4.65 ± 0.81 b |
| TRE | 2.58 ± 0.41 c | 4.54 ± 0.62 f | 4.80 ± 0.76 de | 4.88 ± 0.67 de | 4.97 ± 0.84 bc |
| ROS | 2.40 ± 0.38 bc | 3.86 ± 0.56 def | 3.96 ± 0.63 cd | 2.12 ± 0.36 bc | 1.52 ± 0.22 a |
| ROM | 1.48 ± 0.25 a | 2.76 ± 0.44 bc | 2.92 ± 0.46 bc | 1.78 ± 0.29 ab | 1.10 ± 0.21 a |
| SYL | 4.00 ± 0.61 d | 3.96 ± 0.61 ef | 4.78 ± 0.72 de | 2.77 ± 0.46 c | 1.43 ± 0.26 a |
| ISA | 2.26 ± 0.46 abc | 1.24 ± 0.20 a | 1.27 ± 0.22 a | 1.30 ± 0.19 ab | 1.26 ± 0.23 a |
| SOL | 3.55 ± 0.59 d | 2.29 ± 0.39 b | 1.88 ± 0.32 ab | 1.00 ± 0.17 a | 1.16 ± 0.19 a |

| **HPI** | **CHA** | **ALE** | **CAN** | **TRE** | **ROS** | **ROM** | **SYL** | **ISA** | **SOL** |
| --- | --- | --- | --- | --- | --- | --- | --- | --- | --- |
| 0 | 3.41 ± 0.56 a | 1.74 ± 0.27 a | 2.06 ± 0.40 a | 2.58 ± 0.41 a | 2.40 ± 0.38 b | 1.48 ± 0.25 ab | 4.00 ± 0.61 c | 2.26 ± 0.46 b | 3.55 ± 0.59 c |
| 16 | 3.49 ± 0.55 a | 3.46 ± 0.58 b | 3.00 ± 0.47 a | 4.54 ± 0.62 b | 3.86 ± 0.56 c | 2.76 ± 0.44 c | 3.96 ± 0.61 c | 1.24 ± 0.20 a | 2.29 ± 0.39 b |
| 24 | 6.30 ± 0.86 b | 5.14 ± 0.63 cd | 4.62 ± 0.73 b | 4.80 ± 0.76 b | 3.96 ± 0.63 c | 2.92 ± 0.46 c | 4.78 ± 0.72 c | 1.27 ± 0.22 a | 1.88 ± 0.32 b |
| 48 | 5.77 ± 0.83 b | 4.22 ± 0.70 bc | 4.67 ± 0.71 b | 4.88 ± 0.67 b | 2.12 ± 0.36 ab | 1.78 ± 0.29 b | 2.77 ± 0.46 b | 1.30 ± 0.19 a | 1.00 ± 0.17 a |
| 72 | 6.66 ± 0.98 b | 5.92 ± 0.87 d | 4.65 ± 0.81 b | 4.97 ± 0.84 b | 1.52 ± 0.22 a | 1.10 ± 0.21 a | 1.43 ± 0.26 a | 1.26 ± 0.23 a | 1.16 ± 0.19 a |

**Table S8 - continued**

***VvCHS3***

| **Gen** | **0 HPI** | **16 HPI** | **24 HPI** | **48 HPI** | **72 HPI** |
| --- | --- | --- | --- | --- | --- |
| CHA | 2.78 ± 0.45 bc | 7.96 ± 1.17 d | 8.25 ± 1.04 d | 9.98 ± 1.19 d | 11.69 ± 1.71 d |
| ALE | 2.13 ± 0.32 ab | 5.64 ± 0.75 c | 8.31 ± 1.05 d | 7.94 ± 0.93 c | 9.53 ± 0.95 c |
| CAN | 2.82 ± 0.43 bc | 6.42 ± 0.86 c | 6.27 ± 0.91 c | 8.90 ± 0.81 cd | 10.28 ± 1.39 cd |
| TRE | 3.54 ± 0.63 c | 5.57 ± 0.87 c | 7.56 ± 1.19 cd | 9.83 ± 1.38 d | 10.46 ± 1.19 cd |
| ROS | 2.80 ± 0.36 bc | 4.03 ± 0.59 b | 4.20 ± 0.58 b | 3.32 ± 0.51 b | 2.85 ± 0.49 b |
| ROM | 1.64 ± 0.24 a | 2.99 ± 0.49 ab | 3.14 ± 0.46 b | 1.87 ± 0.31 a | 1.79 ± 0.32 ab |
| SYL | 3.07 ± 0.51 c | 3.05 ± 0.41 ab | 4.33 ± 0.52 b | 1.00 ± 0.19 a | 1.03 ± 0.25 a |
| ISA | 3.18 ± 0.53 c | 1.84 ± 0.47 a | 1.23 ± 0.32 a | 1.38 ± 0.30 a | 1.28 ± 0.31 ab |
| SOL | 3.23 ± 0.43 c | 2.52 ± 0.36 a | 1.18 ± 0.26 a | 1.07 ± 0.22 a | 1.02 ± 0.23 a |

| **HPI** | **CHA** | **ALE** | **CAN** | **TRE** | **ROS** | **ROM** | **SYL** | **ISA** | **SOL** |
| --- | --- | --- | --- | --- | --- | --- | --- | --- | --- |
| 0 | 2.78 ± 0.45 a | 2.13 ± 0.32 a | 2.82 ± 0.43 a | 3.54 ± 0.63 a | 2.80 ± 0.36 a | 1.64 ± 0.24 a | 3.07 ± 0.51 b | 3.18 ± 0.53 b | 3.23 ± 0.43 c |
| 16 | 7.96 ± 1.17 b | 5.64 ± 0.75 b | 6.42 ± 0.86 b | 5.57 ± 0.87 b | 4.03 ± 0.59 bc | 2.99 ± 0.49 b | 3.05 ± 0.41 b | 1.84 ± 0.47 a | 2.52 ± 0.36 b |
| 24 | 8.25 ± 1.04 bc | 8.31 ± 1.05 cd | 6.27 ± 0.91 b | 7.56 ± 1.19 c | 4.20 ± 0.58 c | 3.14 ± 0.46 b | 4.33 ± 0.52 c | 1.23 ± 0.32 a | 1.18 ± 0.26 a |
| 48 | 9.98 ± 1.19 cd | 7.94 ± 0.93 c | 8.90 ± 0.81 c | 9.83 ± 1.38 d | 3.32 ± 0.51 ab | 1.87 ± 0.31 a | 1.00 ± 0.19 a | 1.38 ± 0.30 a | 1.07 ± 0.22 a |
| 72 | 11.69 ± 1.71 d | 9.53 ± 0.95 d | 10.28 ± 1.39 c | 10.46 ± 1.19 d | 2.85 ± 0.49 a | 1.79 ± 0.32 a | 1.03 ± 0.25 a | 1.28 ± 0.31 a | 1.02 ± 0.23 a |
