# Supplementary figures and images for: Transcriptional regulation of stilbene synthases in grapevine germplasm differentially susceptible to downy mildew
Source: BMC Plant Biol. 2019 Sep 14;19:404. doi: 10.1186/s12870-019-2014-5 (PMC6744718; doi:10.1186/s12870-019-2014-5)

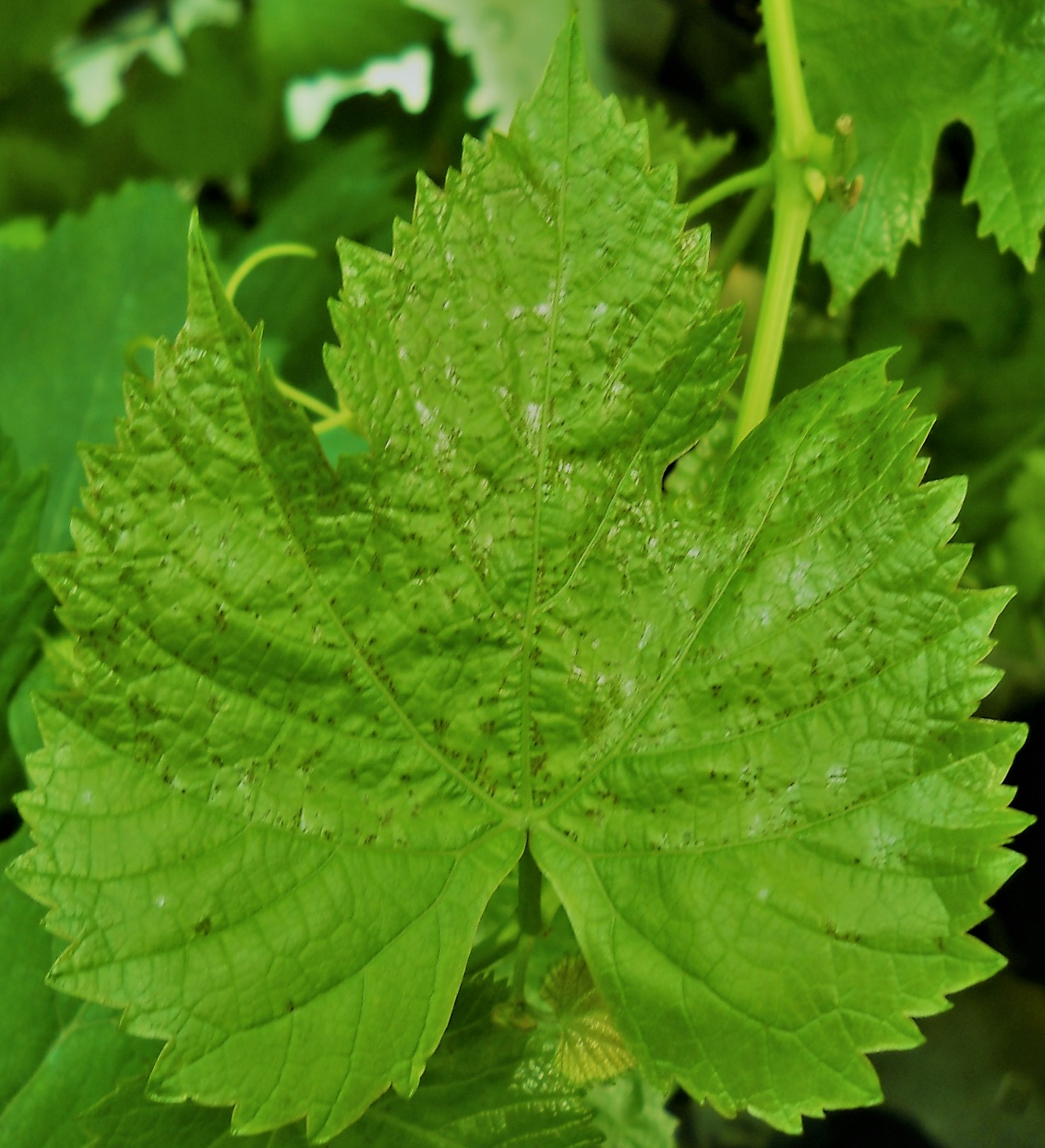

Supplement: Supplementary file 4 — Figure S1. Leaves of the grapevine hybrid Solaris showing hypersensitive reaction seven days after inoculation with Plasmopara viticola. (TIFF 2066 kb) [file 12870_2019_2014_MOESM4_ESM.tiff]

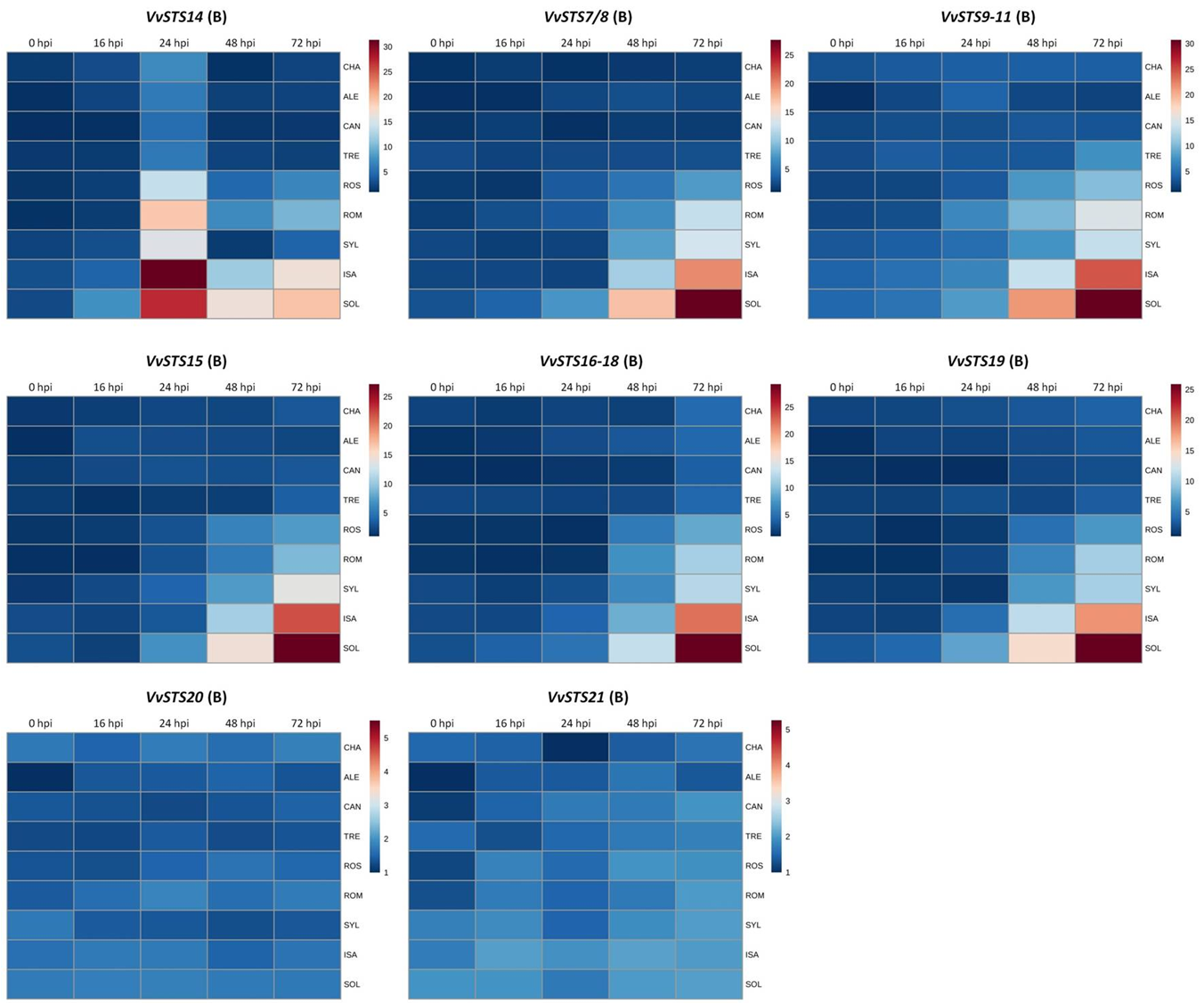

Supplement: Supplementary file 5 — Figure S2. Heat maps of the relative expression levels of the whole set of the Vitis vinifera stilbene synthase genes (VvSTS) in the leaves of the nine grapevine genotypes of Fig. 1 collected up to 72 h after inoculation with Plasmopara viticola. The letter denoting the phylogenetic group to which each gene belongs is given in parentheses. For each gene, relative expression levels were calculated by setting a value of 1 for the lowest value among the nine genotypes in each of the five time points considered. Normalization and sample replication as in Fig. 3. For each gene, the differences in the relative expression levels were shown in color according to the scale and statistical evaluation of the differences among the nine grapevine genotypes and among the five sampling times is reported in Table S6. (ZIP 3113 kb) [file 12870_2019_2014_MOESM5_ESM.zip › Figure S2 continued.tif]

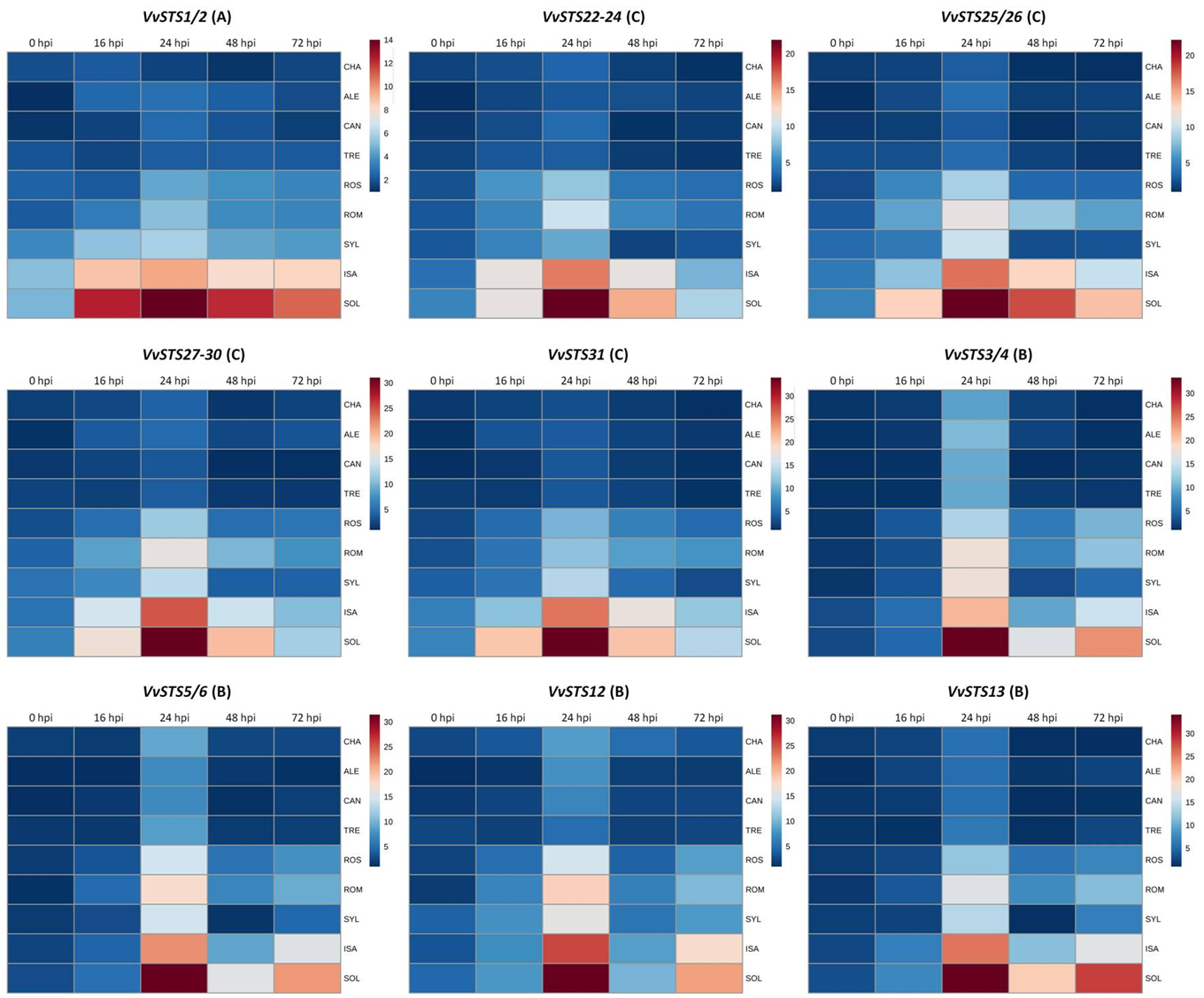

Supplement: Supplementary file 5 — Figure S2. Heat maps of the relative expression levels of the whole set of the Vitis vinifera stilbene synthase genes (VvSTS) in the leaves of the nine grapevine genotypes of Fig. 1 collected up to 72 h after inoculation with Plasmopara viticola. The letter denoting the phylogenetic group to which each gene belongs is given in parentheses. For each gene, relative expression levels were calculated by setting a value of 1 for the lowest value among the nine genotypes in each of the five time points considered. Normalization and sample replication as in Fig. 3. For each gene, the differences in the relative expression levels were shown in color according to the scale and statistical evaluation of the differences among the nine grapevine genotypes and among the five sampling times is reported in Table S6. (ZIP 3113 kb) [file 12870_2019_2014_MOESM5_ESM.zip › Figure S2.tif]
